# PRC2 Is Dispensable *in Vivo* for β-Catenin-Mediated Repression of Chondrogenesis in the Mouse Embryonic Cranial Mesenchyme

**DOI:** 10.1534/g3.117.300311

**Published:** 2017-12-09

**Authors:** James Ferguson, Mahima Devarajan, Gregg DiNuoscio, Alina Saiakhova, Chia-Feng Liu, Veronique Lefebvre, Peter C. Scacheri, Radhika P. Atit

**Affiliations:** * Department of Biology, Case Western Reserve University, Cleveland, Ohio 44106; † Department of Genetics and Genome Sciences, Case Western Reserve University, Cleveland, Ohio 44106; ‡ Department of Cellular and Molecular Medicine, Cleveland Clinic Lerner Research Institute, Ohio 44195; § Department of Dermatology, Case Western Reserve University, Cleveland, Ohio 44106

**Keywords:** cell fate selection, H3K27me3, skull bone, cranial dermis, development

## Abstract

A hallmark of craniofacial development is the differentiation of multiple cell lineages in close proximity to one another. The mouse skull bones and overlying dermis are derived from the cranial mesenchyme (CM). Cell fate selection of the embryonic cranial bone and dermis in the CM requires Wnt/β-catenin signaling, and loss of β-catenin leads to an ectopic chondrogenic cell fate switch. The mechanism by which Wnt/β-catenin activity suppresses the cartilage fate is unclear. Upon conditional deletion of β-catenin in the CM, several key determinants of the cartilage differentiation program, including *Sox9*, become differentially expressed. Many of these differentially expressed genes are known targets of the Polycomb Repressive Complex 2 (PRC2). Thus, we hypothesized that PRC2 is required for Wnt/β-catenin-mediated repression of chondrogenesis in the embryonic CM. We find that β-catenin can physically interact with PRC2 components in the CM *in vivo*. However, upon genetic deletion of Enhancer of Zeste homolog 2 (EZH2), the catalytic component of PRC2, chondrogenesis remains repressed and the bone and dermis cell fate is preserved in the CM. Furthermore, loss of β-catenin does not alter either the H3K27me3 enrichment levels genome-wide or on cartilage differentiation determinants, including *Sox9*. Our results indicate that EZH2 is not required to repress chondrogenesis in the CM downstream of Wnt/β-catenin signaling.

Embryonic craniofacial development involves the formation of complex structures from two progenitor stem cell pools: the cranial neural crest (CNC) and cephalic paraxial mesoderm (PM) (Jiang *et al.* 2002; Yoshida *et al.* 2008). The CNC gives rise to the more anterior tissues of the head and the PM gives rise to the posterior tissues. Both the CNC and PM contribute to the mesenchymal stem cells surrounding the brain called the CM. The cranial bones and the overlying dermis are derived from the CM (Yoshida *et al.* 2008; Tran *et al.* 2010). For proper patterning and development of these tissues, specific signaling pathways are required to regulate cell fate selection and tissue morphogenesis (Fan *et al.* 2016). Disruption of these pathways can lead to craniofacial malformations such as craniosynostosis and focal dermal hypoplasia (Wilkie 1997; Wang *et al.* 2007; Hsu *et al.* 2010). Thus, understanding the genetic mechanisms directing skull and dermal cell fate selection is critical to elucidating the etiology of such malformations.

The Wnt/β-catenin pathway is instructive for cranial bone and dermal fibroblast cell fate selection in the developing embryo (Atit *et al.* 2006; Ohtola *et al.* 2008; Tran *et al.* 2010; Goodnough *et al.* 2012; Fan *et al.* 2016). Conditional loss of ectodermal Wnt-ligand secretion or mesenchymal β-catenin leads to a loss of cranial bones and dermis. Instead, an ectopic formation of cartilage, marked by the upregulation of a key chondrocyte marker gene *Sox9*, is observed (Tran *et al.* 2010; Goodnough *et al.* 2012, 2014; Budnick *et al.* 2016). In intramembranous bone, loss-of-function mutations in other known signaling pathways important in craniofacial development, such as those driven by Fibroblast Growth Factors and Bone Morphogenetic Proteins, do not result in ectopic chondrogenesis (O’Rourke and Tam 2002; Fan *et al.* 2016). In craniofacial development, Wnt/β-catenin signaling seems to have a unique role in the repression of chondrogenesis in the CM.

β-catenin is a central transducer of the canonical Wnt signaling pathway, where it acts as a transcriptional coactivator of context-specific target genes to regulate cell fate selection in many cell types during development (Bhanot *et al.* 1996; Korinek *et al.* 1998; Liu *et al.* 1999; Haegele *et al.* 2003; Verani *et al.* 2007). While β-catenin is typically known as a transcriptional activator, a stabilized or post-translationally methylated form of β-catenin has been shown to function as a transcriptional repressor *in vitro* (Delmas *et al.* 2007; Hoffmeyer *et al.* 2017). However, the mechanism by which Wnt/β-catenin signaling in the CM prevents chondrogenesis, while ensuring proper cranial bone and dermal fibroblast cell fate selection *in vivo*, is unknown.

Recent *in vitro* studies have suggested epigenetic histone modifications, by the PRC2 specifically, as a possible mechanism by which Wnt/β-catenin signaling represses chondrogenesis. PRC2 is a multi-protein complex that is required for the repressive histone modification H3K27me3 (Jiang *et al.* 2002; Lund and Van Lohuizen 2004; Peng *et al.* 2009). In multiple cell types and organisms, numerous connections between the Wnt/β-catenin pathway and PRC2 have been demonstrated. First, like Wnt/β-catenin signaling, PRC2 is required for the regulation of cell fate selection (Lee *et al.* 2006; Sparmann and van Lohuizen 2006; Asp *et al.* 2011; Margueron and Reinberg 2011). Second, *Sox9* and other chondrocyte differentiation determinants are known targets of PRC2 by H3K27me3 enrichment in multiple cell types ranging from mouse embryonic stem cells (ESCs) to chick limb bud micromass cultures (Peng *et al.* 2009; K. I. Kim *et al.* 2013; Kumar and Lassar 2014; Tien *et al.* 2015). Third, PRC2 regulates components of the Wnt/β-catenin pathway and vice versa (Wang *et al.* 2010; Zemke *et al.* 2015; Mirzamohammadi *et al.* 2016; Yi *et al.* 2016). Fourth, β-catenin can physically interact with PRC2 components (Shi *et al.* 2007; Li *et al.* 2009; Jung *et al.* 2013; Hoffmeyer *et al.* 2017). Fifth, β-catenin and PRC2 can cooperate with one another to enhance either Wnt signaling or PRC2 activity (Shi *et al.* 2007; Jung *et al.* 2013; Kumar and Lassar 2014; Hoffmeyer *et al.* 2017). It is important to note that these studies were all performed in cell culture models with one or more overexpressed proteins. Follow-up studies *in vivo* are therefore required. Understanding how Wnt/β-catenin signaling intersects with PRC2 to direct cell fate selection *in vivo* will provide new insights into the genetic mechanisms governing cranial bone and dermal development.

Here, we test the hypothesis that repression of chondrogenesis in the CM by Wnt/β-catenin signaling requires PRC2-mediated epigenetic repression. In a conditional β-catenin loss-of-function model, among the genes dysregulated in both mutant CM and mutant dorsal mesenchyme, we found an overrepresentation of known targets of the PRC2 pathway. Conditional deletion of *Ezh2* in the CM does not phenocopy the ectopic cartilage in the β-catenin mutants, nor do H3K27me3 levels change upon complete loss of β-catenin in the CM. Our results suggest that the repression of chondrogenesis in the CM is not reliant on PRC2, indicating that repressive mechanisms besides PRC2 are likely involved. We propose that the “off” state of chondrogenic genes is not actively maintained by PRC2 and that β-catenin represses chondrogenesis by regulating an unidentified inhibitory pathway.

## Materials and Methods

### Mice and genotyping

The following strains were used in this study: *Engrailed1Cre* (*En1Cre*) (Kimmel *et al.* 2000), *Rosa26 Report*er (*R26R*) (Soriano 1999), β*-catenin* null (β*-catenin*^∆^) (Brault *et al.* 2001), conditional β*-catenin* floxed (β*-catenin^fl^*) (Haegel *et al.* 1995), *Twist2Cre*(*Dermo1Cre*) (Yu *et al.* 2003), and conditional *Ezh2* floxed (*Ezh2^fl^*) (Shen *et al.* 2008). Mice were maintained in mixed genetic backgrounds. For timed matings, *En1Cre*;β*-catenin^+/^*^∆^ males were crossed with *R26R/R26R*;β*-catenin^fl/fl^* females, and *Dermo1Cre*;*Ezh2^fl/+^* males were crossed with *Ezh2^fl/fl^* females. Vaginal plugs were checked every morning and assigned as embryonic (E) 0.5. For each experiment, a minimum of three mutants with litter-matched controls were studied unless otherwise noted. Animals of both sexes were randomly assigned to all the studies. The Case Western Reserve University (CWRU) Institutional Animal Care and Use Committee approved all animal procedures in accordance with AVMA guidelines (Protocol 2013-0156, approved November 21, 2014, Animal Welfare Assurance No. A3145-01).

### CM isolation

At E13.5, the CM was isolated by manual dissection. An incision was made around the circumference of the CM and the tissue covering the brain was manually dissociated. The CM samples were a mixed cell population comprised of the CNC- and PM-derived CM, which is *En1Cre*-positive, and also contained the overlying ectoderm, which is negative for *En1Cre* (CM+ectoderm). Each embryo yielded ∼500,000 CM cells for the controls and 250,000–500,000 CM cells for the mutants. Individual embryos were kept separate and considered as single biological replicates. The wild-type samples isolated for co-immunoprecipitation were dissociated by incubating the tissue in 0.25% Trypsin-EDTA (Thermo Fisher Scientific 25200056) at 37° for 5–7 min, and the CM was selectively enriched from the ectoderm using an Invitrogen FlowComp Flexi Kit (Invitrogen 11060D) and a PDGFRα antibody (5–10 µg/2.5 million cells) (R&D Systems AF1062) (Goodnough *et al.* 2016) according to the manufacturer’s guidelines.

### RT-qPCR

The CM+ectoderm was manually dissected from E13.5 embryos (described above). RNA was isolated as previously described (Hamburg-Shields *et al.* 2015). Relative mRNA expression levels were quantified using 5 ng of cDNA on a StepOne Plus Real-Time PCR System (Life Technologies) and the ΔΔCT method. Commercially available TaqMan probes (Life Technologies) specific to each gene were used: *Ezh2* (Mm00468464_m1), *Suz12* (Mm01304152_m1), *Eed* (Mm00469660_m1), *Sox9* (Mm00448840_m1), *Axin2* (Mm00443610_m1) *Twist2* (Mm00492147_m1), *Keratin14* (mm00516876_m1), and *Pdgfr*α (Mm00440701_m1). CT values obtained for specific genes were normalized to those of β*-actin* (Invitrogen 4352663).

### Immunofluorescence

Heads of E13.5 embryos were fixed in 4% paraformaldehyde for 30 min at 4° and cryopreserved as previously described (Atit *et al.* 2006). Rabbit polyclonal antibodies against H3K27me3 (1:1000; Cell Signaling 9733), LEF1 (1:100; Cell Signaling 2286), SP7/OSX (1:1000; Abcam ab94744), and SOX9 (1:1000; Millipore ab5535) were used for indirect immunofluorescence assays. Appropriate species-specific Alexafluor 594 secondary antibodies were used (1:500; Invitrogen). Images were captured using an Olympus BX60 microscope and an Olympus DP70 digital camera using DC controller software. Confocal images were captured on a Leica TCS SP8 (Leica Biosystems) using Application Suite X software (Leica Biosystems). Images were processed using ImageJ/Fiji (Schindelin *et al.* 2012; Schneider *et al.* 2012) and Adobe Photoshop software. Images were prepared for cell counting in ImageJ/Fiji by subtracting the background and thresholding the signal across all replicates. The percent of the cells that were H3K27me3-positive compared to DAPI was determined using the “analyze particles” feature in ImageJ/Fiji. Counting was performed on the supraorbital CM directly above the eye.

### Co-immunoprecipitation

E13.5 CM was collected by manual dissection and the CM was enriched from the ectoderm (described above). At least 2 million cells from multiple embryos were incubated with 1 ml lysis buffer (50 mM Tris pH 7.5, 250 mM NaCl, 2 mM EGTA, and 1% Triton X-100 in H_2_O) and disrupted with a 23-gauge needle and syringe. The precipitating antibody was added to the lysate and incubated overnight at 4°. Immunoprecipitation was performed by incubating 100 µl of Dynabeads Protein G beads (Invitrogen 10003D) with the lysate and antibody for 1 hr at 4°. Beads were then washed four times with 1 ml wash buffer (40 mM HEPES, 300 mM NaCl, 10% Glycerol, and 0.2% NP40 in H_2_O). The sample was collected in NuPage LDS Sample Buffer (Thermo Fisher Scientific NP0007) supplemented with β-mercaptoethanol, and was heated at 90° for 5 min. Rabbit polyclonal antibodies against nonphospho-β-catenin, 3.43 µg (Cell Signaling D13A1 #8814); EZH2, 10 µg (Diagenode C15410039); and IgG, 6 µg (Abcam ab46540) were used for immunoprecipitation. Protein species were separated by SDS-PAGE using Mini-PROTEAN TGC gels (Bio-Rad #456-1084). Western blots were performed with primary antibodies against β-catenin (1:1000; Millipore 06-734) or EZH2 (1:500, Cell Signaling #5246). Clean-Blot IP Detection Reagent (HRP) (1:250; Thermo Fisher Scientific 21232) was used as secondary antibody.

### Protein isolation and immunoblotting

E13.5 CM+ectoderm was collected by manual dissection. Protein was isolated using RIPA buffer (Cell Signaling 9806). Protein species were separated by SDS-PAGE using Mini-PROTEAN TGC gels (Bio-Rad #456-1084). Western blots were performed with polyclonal rabbit primary antibodies against H3K27me3 (1:1000, Cell Signaling 9733), EZH2 (1:500, Cell Signaling #5246), and SUZ12 (1:1000, Cell Signaling 3737). Species-specific HRP-conjugated secondary antibodies were used at a 1:10,000 dilution. Immunoblots were probed with a rabbit anti-β-tubulin antibody (1:400, Santa Cruz 9104) as a loading control. Signals were detected using an Amersham ECL Western Blotting Analysis System (GE Healthcare RPN2109), and imaged using an Odyssey FC Imaging System (Li-Cor). Relative protein levels were quantitated using ImageJ/Fiji software (Schindelin *et al.* 2012; Schneider *et al.* 2012).

### RNA sequencing

E13.5 CM+ectoderm was collected by manual dissection (described above). Total RNA was isolated from individual embryos as previously described (Hamburg-Shields *et al.* 2015). Libraries were prepared in the CWRU Genomics sequencing core using the Illumina TruSeq Stranded Total RNA kit-with Ribo Zero Gold. Paired-end sequencing was performed on an Illumina HiSequation 2500 v2 Rapid Run flow cell. The resulting 100 bp reads were aligned to the mouse mm9 assembly using TopHat (Trapnell *et al.* 2009; Kim and Salzberg 2011; Langmead and Salzberg 2012; D. Kim *et al.* 2013). Genomic assembly was completed using Cufflinks v1.3 (Trapnell *et al.* 2010, 2013, Roberts *et al.* 2011a,b). mm9_reFlat was used to annotate the data with a maximum intron length of 20,000 bp and genomic bias correction. Cufflinks FPKMs < 0.3 were floored to 0.3. Differential gene expression was determined with CuffDiff using the default settings plus genomic bias correction. Gene ontology analysis examining all differentially expressed genes was performed using Genomic Regions Enrichment of Annotations Tool (GREAT) by associating reads to the single nearest gene located within 5 kb (McLean *et al.* 2010).

### ChIP-seq

E13.5 CM+ectoderm was manually dissected from three *En1Cre*;β*-catenin^fl/+^* and four *En1Cre*;β*-catenin^fl/^*^∆^ embryos, pooled, and H3K27me3 immunoprecipitation and sequencing was performed by Active Motif (www.activemotif.com) (deposited in GEO, GSE96872). Next, 14 µg chromatin was immunoprecipitated with 4 µg rabbit anti-H3K27me3 (Millipore #07-449). Sequencing was performed on an Illumina NextSequation 500 producing 75-nucleotide, single-end reads. *Drosophila* DNA was “spiked in.” The ratio of aligned *Drosophila* reads in the mutant *vs.* control samples (calculated to be 1.3) was used to normalize the number of reads in the mouse samples by downsampling the larger sample (mutant, in this case). Sequences were aligned and analyzed twice independently. The analysis was first performed using a custom pipeline consisting of Bowtie2 for genome alignment to the mouse mm9 genome and Macs 1.4 at default settings for peak calling (Zhang *et al.* 2008; Langmead and Salzberg 2012). To generate the windowed heat map from this analysis, the genome was divided into 40 windows of equal size 5 kb up- and downstream of each H3K27me3 peak genome-wide or on peaks located within 1 kb of known promoters. The median peak signal in each window was then converted to a z-score and mapped using Java TreeView (Saldanha 2004). The analysis was performed a second time using the NGS 2.8 pipeline (Strand NGS Manual, Version 2.8, Build 230243, Strand Life Sciences, Bangalore, India) and aligning to the mm10 genome. Peaks were called using Macs 1.4 at default settings. Association of peaks with specific genes was performed using PAVIS (Huang *et al.* 2013). Specific H3K27me3 peaks were visualized using the Integrated Genome Viewer (IGV) (Robinson *et al.* 2011; Thorvaldsdóttir *et al.* 2013). Ngs.plot was used to generate the average fold enrichment of H3K27me3 overview across the gene bodies (Shen *et al.* 2014).

### Cell culture

The CM+ectoderm was manually isolated and dissociated by incubating the tissue in 0.25% Trypsin-EDTA (Thermo Fisher Scientific 25200056) at 37° for 5–7 min, and then plated in DMEM with 10% fetal bovine serum. Fibroblasts were allowed to adhere to the plate for 1–2 hr, after which the media was removed and fresh media was added. Chemical inhibition was performed at no later than passage 3. Next, 10% Wnt3a-conditioned media and the chemical inhibitor, UNC1999 (Sigma SML0778) or GSK126 (Cayman Medical CAS1346574-57-9), were added simultaneously. The cells were incubated for the indicated amount of time. Following incubation, the cells were trypsinized and processed for protein or mRNA analysis.

### Statistics

Graphs and statistical analyses were generated using Prism 6 (GraphPad Software). Data are presented as mean ± SEM in all graphs unless otherwise stated. All pairwise sample comparisons were performed using a Mann–Whitney test. The *P*-values for statistical tests in all figures are represented as * *P* < 0.05 and ** *P* < 0.01.

### Data availability

Strains are publicly available at the Jackson Laboratory. Sequencing data are available at GEO with the accession number GSE96872.

## Results

### Genes dysregulated upon loss of β-catenin are enriched for the PRC2-associated H3K27me3 histone mark

**Figure 1:**
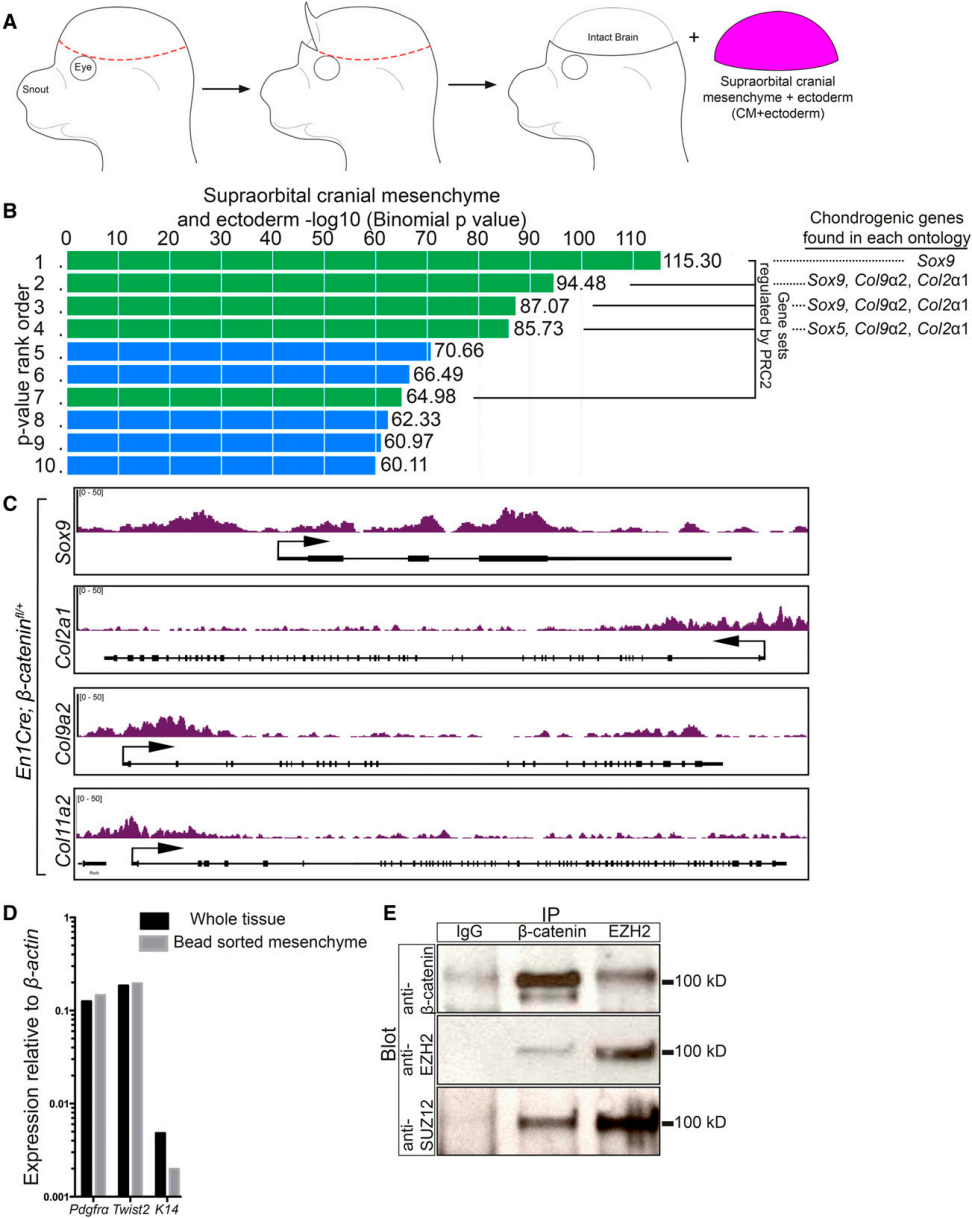
β-catenin regulates known PRC2 targets and can physically interact with PRC2. (A) Schematic demonstrating the isolation of CM+ectoderm by manual dissection. The isolated tissue is comprised of the supraorbital CM and overlying ectoderm. (B) Gene ontology analysis of differentially expressed genes in E13.5 *En1Cre/*+;*R26R/*+;β*-catenin^fl/^*^+^ control (*n* = 5) and *En1Cre/*+;*R26R/*+;β*-catenin^fl/^*^∆^ mutant CM+ectoderm (*n* = 4). Overlapping gene sets from MsigDB Perturbations ontology ranked by binomial *P*-value, which accounts for varying sizes of gene regulatory domains, from GREAT. Green bars represent gene sets associated with PRC2. The ranked list is as follows: (1) Genes with high-CpG-density promoters bearing H3K4me2 and H3K27me3 in the brain (MsigDB M1941). (2) Genes with H3K27me3 on their promoters in human embryonic stem cells identified by ChIP on chip (MsigDB M10371). (3) Genes identified as targets of SUZ12 in human embryonic stem cells by ChIP on chip (MsigDB M2291). (4) Genes identified as targets of EED in human embryonic stem cells by ChIP on chip (MsigDB M2736). (5) Genes coordinately upregulated in a compendium of adult tissue stem cells (M1999). (6) Genes upregulated in uterus upon knockout of BMP2 in the uterus (MsigDB M2324). (7) Genes downregulated in mouse embryonic stem cells upon deletion of SUZ12 (MsigDB M2291). (8) Genes upregulated in human lung fibroblasts (IMR90) after knockdown of RB1 by RNAi (MsigDB M2129). (9) Genes downregulated in breast cancer (MsigDB M13072). (10) Genes downregulated in immortalized nontransformed mammary epithelium (HMLE) after knockdown by RNAi or expression of a dominant negative form of CDH1 (MsigDB M11790). (C) Integrated Genome Viewer representation of H3K27me3 ChIP-sequencing on E13.5 *En1Cre/*+;*R26R/*+;β*-catenin^fl/^*^+^ control CM+ectoderm of *Sox9* (chr11:112,641,524–112,651,071), *Col2a1* (chr15:97,804,033–97,837,155), *Col9a2* (chr4:120,710,171–120,729,930), and *Col11a2* (chr17:34,174,382–34,205,187). (D) RT-qPCR of enriched CM after bead purification with an antibody specific to PDGFRα. A reduction in K14 expression levels demonstrates purification of the mesenchyme. (E) Co-immunoprecipitation of β-catenin with components of PRC2. CM+ectoderm was isolated by manual dissection and the mesenchyme was purified using a PDGFRα antibody. IgG was used as a negative immunoprecipitation control. ChIP, chromatin immunoprecipitation; chr, chromosome; CM, cranial mesenchyme; MsigDB, Molecular Signatures Database; PRC2, Polycomb Repressive Complex 2; RNAi, RNA interference; RT-qPCR, reverse transcriptase-quantitative polymerase chain reaction.

In an effort to determine a functional link between β-catenin and PRC2 *in vivo*, we conditionally deleted β-catenin in the CM using *Engrailed1Cre* (*En1Cre*), manually dissected the CM along with the ectoderm (CM+ectoderm), and collected all the CNC- and PM-derived mesenchyme surrounding the brain (Figure 1A) (Kimmel *et al.* 2000; Tran *et al.* 2010). *En1Cre* is expressed in both the CNC- and PM-derived CM. In order to analyze *in vivo* tissues with minimal manipulation, the ectoderm was isolated with the CM. We then profiled the whole transcriptome on three litter-matched E13.5 *En1Cre/+*;*R26R/+*;β*-catenin^fl/+^* controls and four *En1Cre/+*;*R26R/+*;β*-catenin^fl/^*^Δ^ mutants using the RNA-seq approach (GSE96872). The analysis of the data revealed 521 genes that were differentially expressed by at least 1.4-fold in the two experimental groups (*P* < 0.05). Of the 521 differentially expressed genes, 322 were downregulated and 199 were upregulated in the mutants relative to the controls. Validating the approach, changes in expression of known Wnt/β-catenin targets were observed despite the presence of ectodermal cells, in which canonical Wnt signaling is known to be active (Supplemental Material, Figure S1A in File S1 [all Supplemental legends are in File S2] (Budnick *et al.* 2016). To ascertain the function of all the 521 differentially expressed genes, we performed a gene ontology analysis using GREAT, which queries multiple ontology databases (McLean *et al.* 2010). As a comparison, we also analyzed RNA-seq data from E13.5 *En1Cre/+*;*R26R/+*; β*-catenin^fl/^*^Δ^ dorsal dermal mesenchyme (GSE75944) (Budnick *et al.* 2016). The top five ontologies of the differentially expressed genes in both the mutant CM+ectoderm and mutant dorsal dermal fibroblasts included the Wnt signaling pathway, along with Cadherin signaling, Integrin signaling, and ECM-receptor interactions (Figure S1, B and C in File S1) (Thomas *et al.* 2003). In the Molecular Signatures Database (MsigDB) Perturbations ontology, we also found that both data sets were highly enriched for genes regulated by PRC2 (Figure 1B and Figure S2A in File S1) (Subramanian *et al.* 2005). Interestingly, enrichment for targets of PRC2 can be found in both the up- and downregulated genes. However, this enrichment is unique only to the genes differentially expressed in our β-catenin mutants. GREAT analysis on all genes expressed in the CM+ectoderm (FPKM ≥ 1) did not result in enrichment for targets of PRC2 in the MSigDB Perturbations ontology (Figure S2B in File S1). Thus, the differential expression of PRC2 targets in the β-catenin mutant CM+ectoderm and dorsal mesenchyme reveals a potential functional link between the two pathways.

### Chondrocyte fate genes are enriched for H3K27me3 in the embryonic CM

To establish a role for PRC2 in the repression of chondrogenesis in the CM *in vivo*, we queried for H3K27me3 enrichment in the loci of individual chondrocyte marker genes. We manually dissected the CM+ectoderm in E13.5 *En1Cre/+*;*R26R/+*;β*-catenin^fl/+^* controls and performed ChIP using an antibody against H3K27me3 followed by massive parallel DNA sequencing (ChIP-seq). This assay allowed us to unbiasedly and comprehensively map the genome-wide distribution of the H3K27me3 modification (Active Motif Technology) (GSE96872). In the CM+ectoderm of E13.5 controls, the transcriptional start sites of multiple cartilage markers, such as *Sox9*, *Col2a1*, *Col9a2*, and *Col11a2* (Figure 1C), were enriched for H3K27me3, indicating that they are targets of PRC2 in the CM.

### Endogenous β-catenin and EZH2 may physically interact in the CM

Given the emerging connections made between the Wnt/β-catenin pathway and PRC2 in various systems *in vitro* (summarized in Table S1 in File S1), we set out to test the hypothesis that β-catenin and PRC2 components physically interact at native protein levels in the mouse CM extracts. We manually dissected the CM, made a cell suspension, and used a PDGFRα antibody bound to magnetic beads to enrich for the CM population (Goodnough *et al.* 2016). We found comparable levels of mRNA for mesenchyme progenitor markers *Pdgfrα* and *Twist2*, and diminished ectoderm marker *Keratin 14* (*K14*) in the purified sample, confirming enrichment for CM (Figure 1D). We then prepared cell extracts from sorted CM and used them in a co-immunoprecipitation assay for β-catenin and EZH2. EZH2 is the methlytransferase component of PRC2, and is required for the H3K27me3 modification (Margueron and Reinberg 2011). In line with our hypothesis, β-catenin successfully co-immunoprecipitated with the EZH2 antibody. In addition, we also observed reciprocal co-immunoprecipitation of EZH2 and another major PRC2 component, SUZ12, by the β-catenin antibody (Figure 1E). These results suggest that PRC2 components and β-catenin may physically interact at wild-type expression levels in the CM. Thus, these data provide a potential molecular link between Wnt/β-catenin signaling and PRC2 in the mouse embryo.

### β-catenin is not required for PRC2 component expression or bulk H3K27me3 levels

**Figure 2:**
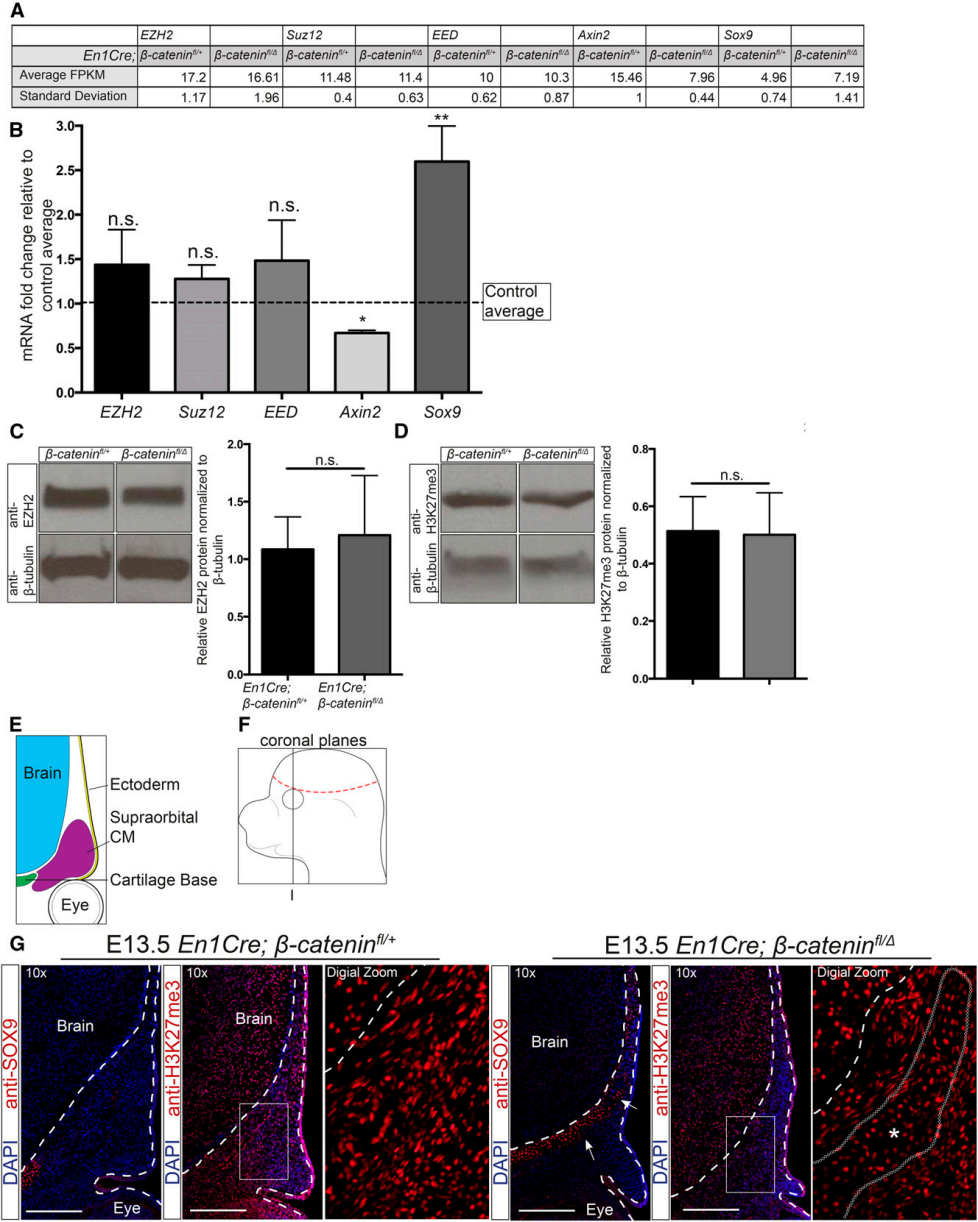
β-catenin activity is not required for the expression of PRC2 components and bulk H3K27me3 levels. (A) FPKM values obtained from RNA sequencing assays in CM+ectoderm from *En1Cre/*+;*R26R/*+;β*-catenin^fl/^*^∆^ mutant and *En1Cre/*+;*R26R/*+;β*-catenin^fl/^*^+^ controls. (B) RT-qPCR analysis of the CM+ectoderm in *En1Cre/*+;*R26R/*+;β*-catenin^fl/^*^+^ (*n* = 7) and *En1Cre/*+;*R26R/*+;β*-catenin^fl/^*^∆^ mutants (*n* = 9). The data are represented as fold change in mutants over controls. The dotted line represents the β-catenin controls. *Axin2* and *Sox9* are known targets regulated by β-catenin. (C and D) Western blot analysis (*n* = 5) of EZH2 and H3K27me3 in CM+ectoderm from *En1Cre/*+;*R26R/*+;β*-catenin^fl/^*^∆^ mutants and *En1Cre/*+;*R26R/*+;β*-catenin^fl/^*^+^ controls. Band intensities were quantified using ImageJ. (E and F) Schematics representing the supraorbital CM in coronal sections near the frontal bone primordia. (G) Indirect immunofluorescence of SOX9, H3K27me3, and DAPI in the supraorbital mesenchyme (*n* = 2 controls; 3 mutants). Images were taken near the frontal bone primordia (plane I). Dashed lines indicate the brain and ectoderm boundaries. (*) indicates region of ectopic cartilage. Bar, 200 µm. CM, cranial mesenchyme; DAPI, 4’,6-diamidino-2-phenylindole; FPKM, fragments per kilobase of transcript per million mapped reads; n.s., not significant; RT-qPCR, reverse transcriptase-quantitative polymerase chain reaction.

To determine if β-catenin is required for the formation of the PRC2 complex itself, we first examined the expression of the main PRC2 components: *Ezh2*, *Suz12*, and *Eed*. Based on FPKM values from our RNA-seq data set, we found no significant changes in the PRC2 component mRNA levels (Figure 2A). To validate this result, we manually dissected E13.5 *En1Cre/+*;*R26R/+*;β*-catenin^fl/+^* control and *En1Cre/+*;*R26R/+*;β*-catenin^fl/^*^Δ^ mutant CM+ectoderm (Figure 1A), and determined the mRNA levels of *Ezh2*, *Suz12*, and *Eed* by RT-qPCR. Similar to the RNA-seq data set, the relative mRNA levels of the individual PRC2 components were comparable in the control and mutant samples (Figure 2B). In comparison, the expected changes in mRNA levels were observed in known β-catenin responsive genes *Axin2* and *Sox9* (Figure 2, A and B) (Jho *et al.* 2002; Goodnough *et al.* 2012). Evaluation of the total H3K27me3 and EZH2 protein levels using western blot assays also revealed comparable protein levels between control and β-catenin mutant CM+ectoderm (Figure 2, C and D). To obtain spatial information and account for levels in the ectoderm between our control and mutants, we performed indirect immunofluorescence for H3K27me3 in the E13.5 *En1Cre/+*;*R26R/+*;β*-catenin^fl/+^* control and *En1Cre/+*;*R26R/+*;β*-catenin^fl/^*^Δ^ mutants (Figure 2, E–G). While we consider the CM to include the entire CM surrounding the brain (Figure 1A), we focused our indirect immunofluorescence analysis on the region directly above the eye (supraorbital CM) (Figure 2E) due to easily identified histological landmarks such as the eye and brain ventricles. Considering that knockout of β-catenin results in ectopic chondrogenesis throughout the CM, we expect the supraorbital CM to be representative of the entire CM. In the supraorbital CM, both the control and β-catenin mutants are positive for H3K27me3, demonstrating that PRC2 is still active without β-catenin. Furthermore, H3K27me3 can still be found in the expanded SOX9 domain in the β-catenin mutants. We concluded that β-catenin is not required cell autonomously in the CM to regulate the relative mRNA levels of major PRC2 components, the EZH2 protein levels, and bulk H3K27me3 levels. However, these results leave open the possibility that it may be required to recruit PRC2 to site-specific loci on the genome.

### Loss of Ezh2 does not lead to ectopic cell type fate selection or chondrogenesis in the CM

**Figure 3:**
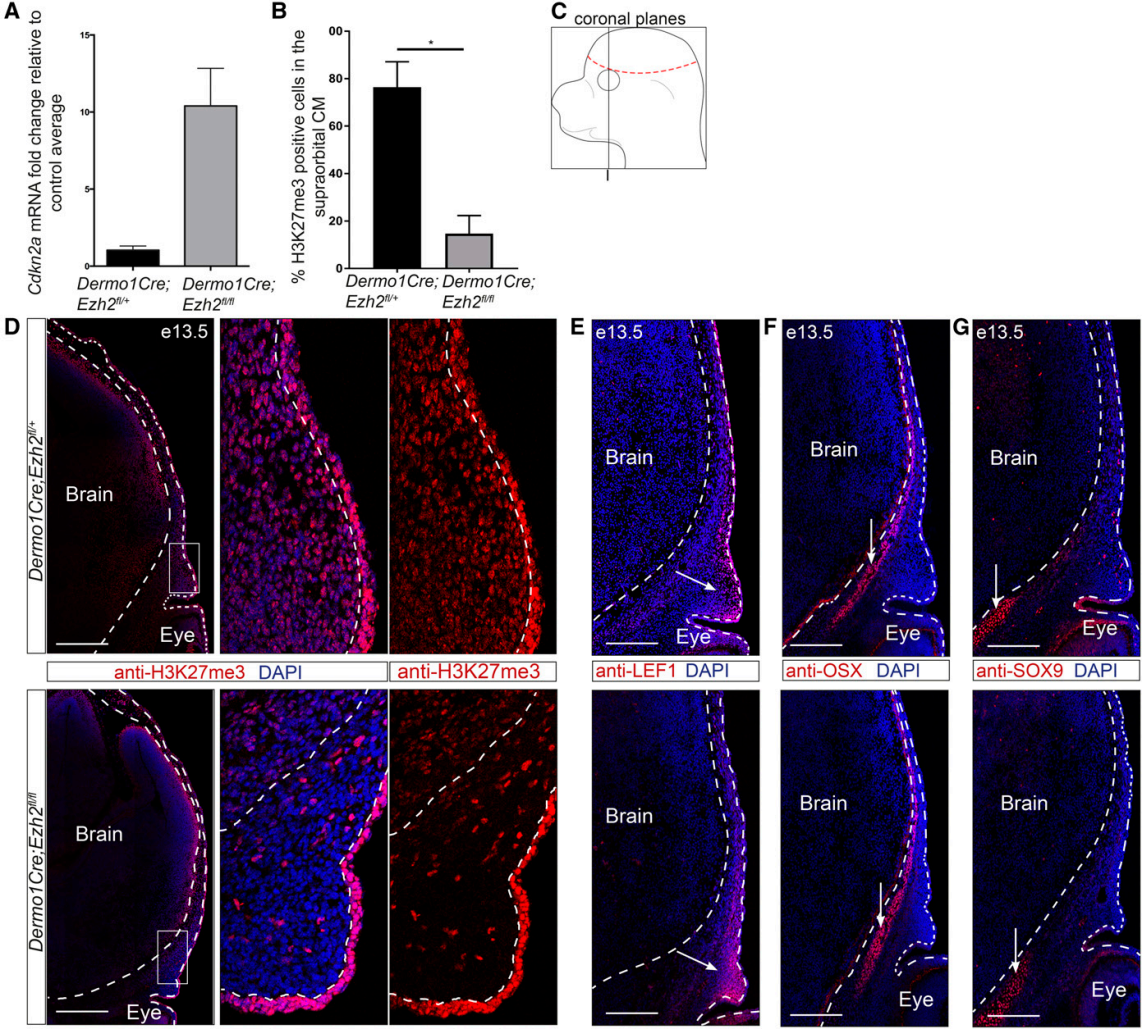
Knockdown of *Ezh2* in the cranial mesenchyme does not lead to changes in cell fate selection. (A) Known downstream target *Cdkn2a* expression relative to controls in *Dermo1Cre*;*Ezh2^fl/fl^* CM+ectoderm (*n* = 2). (B) Percent H3K27me3 positive cells in the supraorbital CM. (C) Schematic representing the coronal sections near the frontal bone primordia. (D–G) Indirect immunofluorescence in *Dermo1Cre*;*Ezh2^fl/fl^* supraorbital CM. (D) Qualitative loss of the PRC2 repressive mark H3K27me3. (E–G) Domains of similar size and location were observed for the osteoblast (OSX), dermal fibroblast (LEF1), and chondrocyte (SOX9) markers between controls and mutants. Dashed lines indicate the brain and ectoderm boundaries. (D–F) Arrows mark the tissue domains. Bar, 200 µm. CM, cranial mesenchyme; DAPI, 4’,6-diamidino-2-phenylindole; PRC2, Polycomb Repressive Complex 2. * *P* < 0.05.

We next determined if PRC2 is required for the repression of chondrogenesis in the CM *in vivo*. In order to remove PRC2 function in the CM, we conditionally deleted *Ezh2* using a floxed allele (Shen *et al.* 2008). Surprisingly, conditional deletion of *Ezh2* at E10.5 using *En1Cre* did not lead to the expected loss of H3K27me3 in the CM by indirect immunofluorescence (Figure S3 in File S1). We then conditionally deleted *Ezh2* in the CM using *Dermo1Cre*, which is expressed in the CM by E10.0 (Yu *et al.* 2003; Goodnough *et al.* 2012). Loss of *Ezh2* was sufficient to lead to an upregulation of *Cdkn2a*, a known target of PRC2 (Figure 3A) (Shen *et al.* 2008; Lui *et al.* 2016). We also found depletion of H3K27me3 in the supraorbital CM (Figure 3C) by indirect immunofluorescence between the *Dermo1Cre*; *Ezh2^fl/fl^* mutants to *Dermo1Cre*; *Ezh2^fl/+^* controls (Figure 3, B and D). H3K27me3 signal was maintained in both the ectoderm and the brain, where *Dermo1Cre* is not expressed. After confirming the absence of PRC2 activity, we then examined the protein level of cell fate markers for bone, dermis, and cartilage progenitors by indirect immunofluorescence. Conditional deletion of *Ezh2* in the supraorbital CM did not lead to changes in the location and size of the dermal domain as indicated by LEF1, and the bone domain as indicated by Osterix (OSX) (Figure 3, E and F). Consistently, we did not observe ectopic expression beyond the cartilage base of the key cartilage differentiation determinant SOX9 (Figure 2G). Based on these data, *Ezh2* has little effect on the patterning of the tissue domains and minimal effect on the protein expression levels by immunofluorescence.

**Figure 4:**
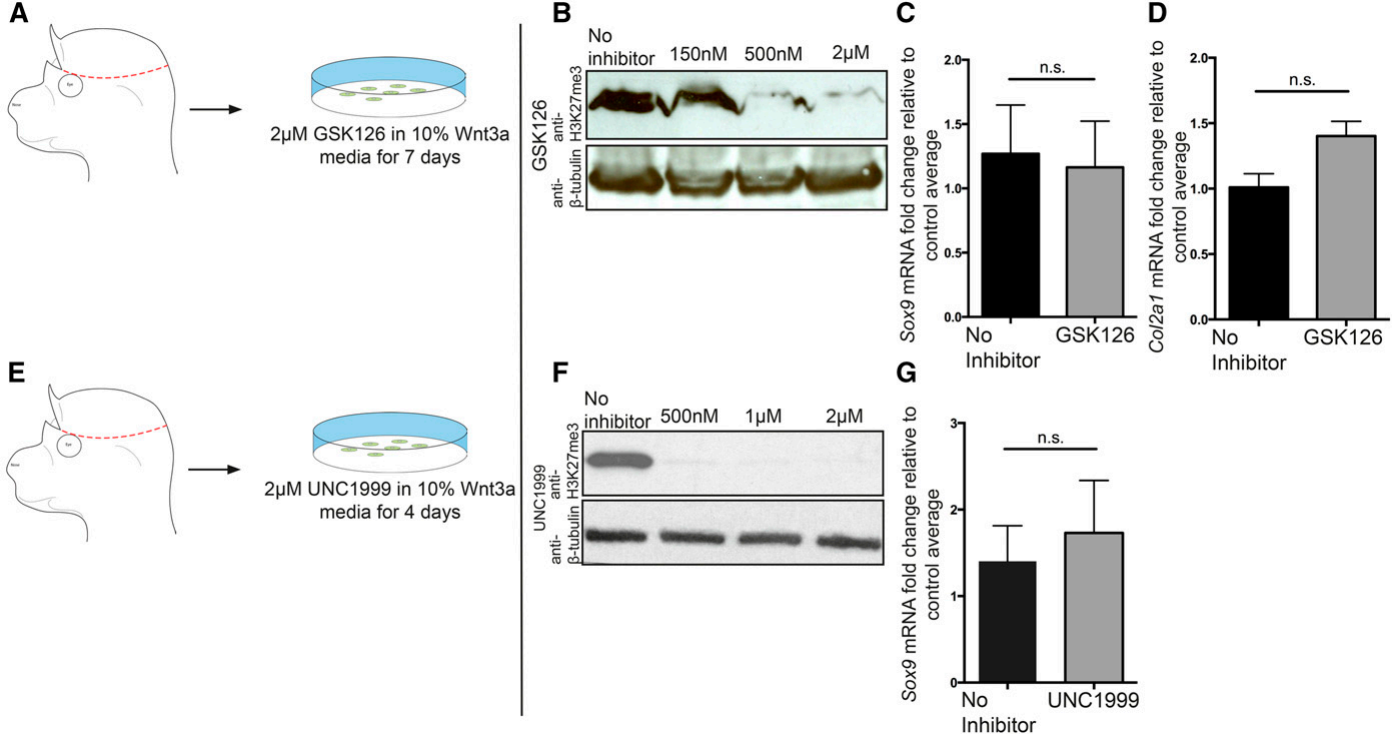
Chemical inhibition of EZH2 methyltransferase does not lead to an upregulation of early chondrocyte markers in CM+ectoderm. (A and E) Schematic demonstrating the isolation of primary CM+ectoderm fibroblasts. GSK126 is specific to EZH2, and UNC1999 is specific to both EZH2 and EZH1. GSK126 and UNC1999 inhibit EZH2’s methyltransferase activity. (B and F) Western blots demonstrating reduction/loss of H3K27me3 level following incubation with GSK126 ((IC50 = 75–100 nm) or UNC1999 (IC_50_ < 10 nM for EZH2 and 45 nM for EZH1). (C, D, and G) qPCR analysis of the expression of *Sox9* and *Col2a1* following inhibition of EZH2. GSK126: *n* = 5 mutants and 6 controls for *Sox9*, and *n* = 3 mutants and controls for *Col2a1*. UNC1999: *n* = 7 mutants and 9 controls. CM, cranial mesenchyme; n.s., not significant; qPCR, quantitative polymerase chain reaction. * *P* < 0.05; ** *P* < 0.01.

To further test the H3K27me3-dependent role of PRC2 in the repression of chondrogenesis, we chemically inhibited EZH2 function in primary E13.5 CM+ectoderm cells *in vitro* (Figure 4, A and E). Incubation with small molecule methyltransferase inhibitor GSK126, which is specific for EZH2, or with UNC1999, which inhibits EZH2 and EZH1, led to a considerable reduction in bulk H3K27me3 protein levels (Figure 4, B and F). Upon treatment with GSK126 or UNC1999, the *Sox9* and *Col2a1* mRNA levels were not significantly increased (Figure 4, C, D, and G). Taken together, these data indicate that EZH2 and H3K27me3 are dispensable for regulating the mRNA level of chondrocyte differentiation markers in the CM+ectoderm.

### Loss of β-catenin does not significantly alter H3K27me3 enrichment genome-wide

**Figure 5:**
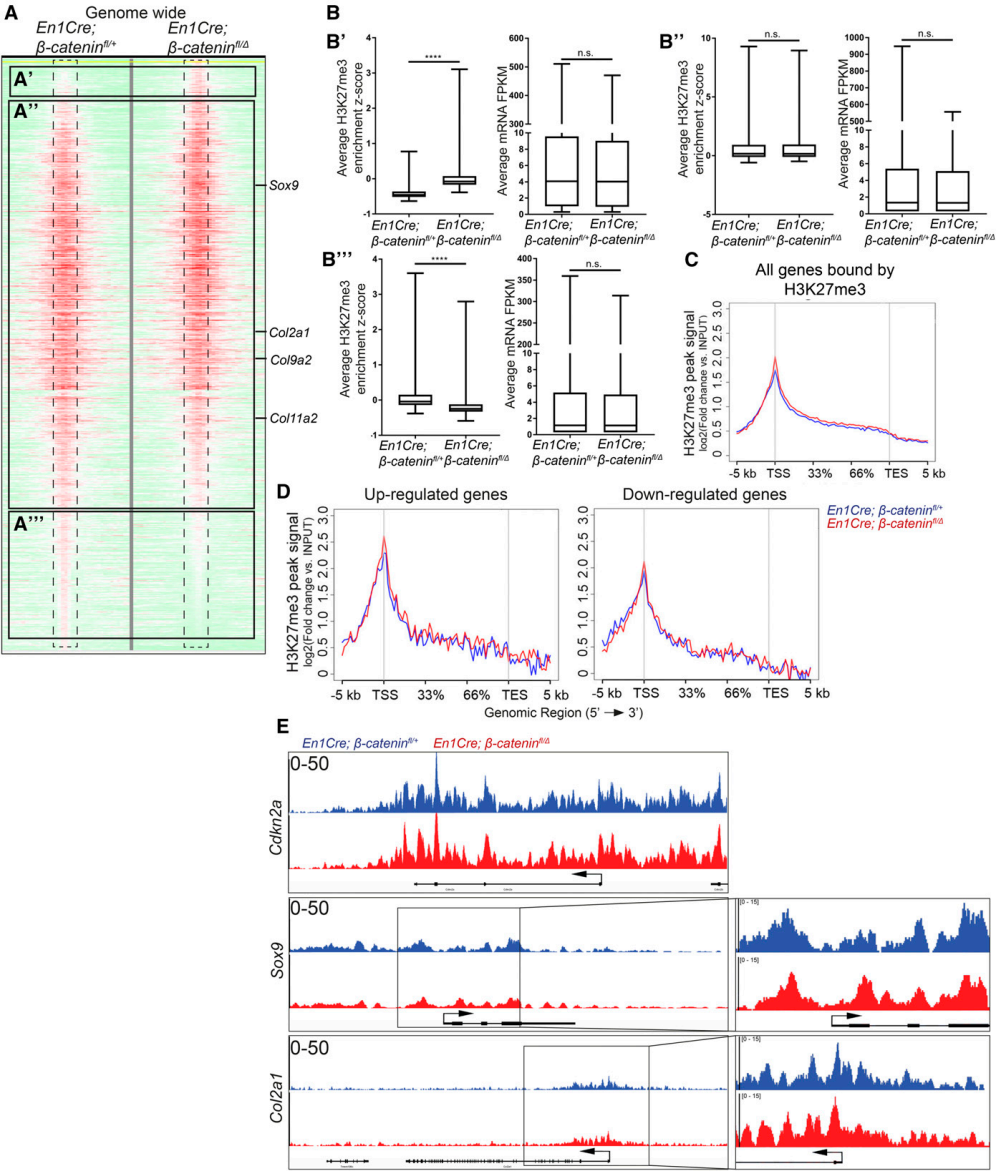
Loss of β-catenin does not significantly alter H3K27me3 enrichment genome-wide or on cartilage differentiation determinants. (A) Treeview representation of H3K27me3 peak strength genome-wide in the CM+ectoderm between E13.5 *En1Cre/*+;*R26R/*+;β*-catenin^fl/^*^+^ controls and *En1Cre/*+;*R26R/*+;β*-catenin^fl/^*^∆^ mutants. H3K27me3 ChIP-sequencing signal strength was mapped 5 kb up- and downstream from each peak. A change from high intensity to low intensity identifies a peak is lost. (B) Intersection of changes in H3K27me3 enrichment from (A), with *En1Cre/*+;*R26R/*+;β*-catenin^fl/^*^∆^ mutant and *En1Cre/*+;*R26R/*+;β*-catenin^fl/^*^+^ control RNA-seq (B’–B’’’) corresponding with (A’–A’’’), respectively. (C and D) Intersection of *En1Cre/*+;*R26R/*+;β*-catenin^fl/^*^+^ control and *En1Cre/*+;*R26R/*+;β*-catenin^fl/^*^∆^ mutant ChIP-sequencing and RNA-seq. H3K27me3 ChIP-sequencing signal strength was measured across all genes bound by H3K27me3 or genes identified to be differentially expressed in β-catenin mutant CM+ectoderm. The *x*-axis demarcates the percent distance across a gene between the TSS and the TES. (E) IGV representation of H3K27me3 signal peaks between *En1Cre/*+;*R26R/*+;β*-catenin^fl/^*^+^ control (*n* = 1) and *En1Cre/*+;*R26R/*+;β*-catenin^fl/^*^∆^ mutant (*n* = 1) CM+ectoderm. *Cdkn2a* is a known target of PRC2. *Sox9* and *Col2a1* are chondrocyte marker genes. ChIP, chromatin immunoprecipitation; CM, cranial mesenchyme; FPKM, fragments per kilobase of transcript per million mapped reads; n.s., not significant; RNA-seq, RNA-sequencing; TES, transcription end site; TSS, transcription start site.

Next, we tested to what extent β-catenin is required for the recruitment of PRC2 to the genome in a site-specific manner. We performed ChIP-seq assays, as described in Figure 1, to map the genome-wide distribution of H3K27me3 in the CM *in vivo* between *En1Cre/+*;*R26R/+*;β*-catenin^fl/+^* controls and *En1Cre/+*;*R26R/+*;β*-catenin^fl/^*^Δ^ mutants (GSE96872). Sequencing of the CM+ectoderm revealed, by two independent analyses, 14,337 peaks in the control and 10,752 peaks in the mutant, thus 25% fewer peaks in the mutant. Surprisingly, genome-wide comparisons between individual mutant and control H3K27me3 peaks revealed modest differences in enrichment fold between the two samples (Figure 5A). The differences in peak numbers between β*-catenin* controls and mutants were associated with changes in smaller H3K27me3 peaks (Figure 5, A’, A’’’, B’, and B’’’). Furthermore, any gains and losses of strength of H3K27me3 peaks were not associated with gene expression changes (Figure 5B). In addition, on all genes bound by H3K27me3, the signal intensity of the peaks was also comparable between the mutant and control across the gene body (Figure 5C). Next, we examined changes in H3K27me3 peak signal across the gene body of the differentially expressed genes identified in our RNA-seq data. In both the up- and downregulated genes, the H3K27me3 enrichment was comparable between β-catenin controls and mutants (Figure 5D).

From our ChIP-seq data set, we observed variations of the H3K27me3 enrichment throughout the genome ranging from large peaks blanketing an entire gene body to smaller peaks located just on the promoter. To further investigate the connection between H3K27me3 peak enrichment strength and gene expression, we divided the H3K27me3 peaks into three categories based on the level of enrichment: strong (>20-fold enrichment), medium (10–20-fold enrichment), and weak (≤ fivefold enrichment) (Figure S4 in File S1). Representative enrichment for strong, medium, and weak peaks can be found on the *HoxA* cluster, *Sept9*, and *Lmtk3*, respectively (Figure S4A in File S1). The genomic location of each class of peak using GREAT revealed that the large majority of strong and medium peaks were within 5 kb of the transcription start site (TSS), and the weak peaks had a more even distribution spanning out 500 kb from the TSS (Figure S4B in File S1). Between controls and β-catenin mutants, the number of strong and medium peaks was comparable, with most of the variation found in the weak peaks (Figure S4C in File S1). To further characterize each class of peak, we performed gene ontology analysis on the control H3K27me3 ChIP-seq data set. Gene ontology analysis revealed distinct functions for the strong peaks such as DNA-binding/transcription regulation and conserved homeobox sites, and the medium and weak peaks shared functions such as Wnt signaling and ion transport (Figure S5 in File S1). Furthermore, comparisons between each class of peak found near a TSS (±5 kb) and the genes identified in our RNA-seq data set revealed that 70% of strong peaks, 53% of medium peaks, and 47% of weak peaks were associated with transcriptional repression (<1 FPKM) (Figure S6A in File S1). These results indicate that the level of H3K27me3 enrichment may be predictive of its transcriptional repressive function. When we intersected genes bound by each class of H3K27me3 peak and differentially expressed genes, we found that each class of peak had similar enrichment between the down- and upregulated genes, indicating that H3K27me3 enrichment does not predict transcriptional repression by β-catenin (Figure S6B in File S1).

### H3K27me3 enrichment is not depleted on ectopically-expressed chondrocytic gene determinants in β-catenin mutants

To determine if the loss of β-catenin resulted in depletion of H3K27me3 on chondrocyte differentiation determinants, we examined the enrichment of H3K27me3 on *Sox9* and its downstream target *Col2a1*, which have higher mRNA levels in the β-catenin mutant CM (Figure 2) (Goodnough *et al.* 2012). *Cdkn2a*, *HoxA*, and *T/Brachyury* are known targets of PRC2, contain strong H3K27me3 peaks, and serve as controls. Upon deletion of β*-catenin*, we did not observe a change in H3K27me3 enrichment on known PRC2 target genes (Figure 5E and Figure S7, A and B in File S1). More importantly, H3K27me3 enrichment did not change on *Sox9*, *Col2a1*, *Col9a2*, or *Col11a2* (Figure 5E and Figure S7, H and I in File S1). Furthermore, H3K27me3 enrichment was similar between β-catenin controls and mutants on the TSS of critical bone and dermal marker genes, such as *Runx2*, *Twist1*, *Twist2*, *Axin2*, and *Lef1* (Figure S7, C–G in File S1). *Mcm6* serves as a negative control and lacks H3K27me3 enrichment in either the control or mutant (Figure S7J in File S1). It is worth noting that *Cdkn2a*, *HoxA*, and *T* loci had strong H3K27me3 enrichment peaks, while *Sox9* and *Col2a1* loci had medium enrichment peaks in both controls and β-catenin mutants (Figure 5E and Figure S7, A and B in File S1). Our results showed that H3K27me3 enrichment is not depleted from the TSS of chondrocyte differentiation determinants in β-catenin mutants and remains enriched in actively transcribed genes.

## Discussion

Based on *in vivo* evidence that β-catenin is required to repress chondrogenesis in the CM, and on emerging *in vitro* evidence connecting β-catenin and PRC2 in other processes, we tested the hypothesis that repression of chondrogenesis by Wnt/β-catenin signaling requires epigenetic repression by PRC2 *in vivo*. Consistent with the findings from previous studies, our results demonstrate that an *in vivo* loss of β-catenin in the CM and dorsal mesenchyme leads to the activation of chondrogenic marker genes such as *Sox9*, *Col2a1*, and *Col11a2*, as well as other known PRC2 target genes. Further, we find that β-catenin can physically interact with PRC2 components at native protein levels in CM-enriched protein extracts. In contrast to findings from *in vitro* studies, we observe that β-catenin is not required for the expression of major PRC2 components *in vivo* and that PRC2 is dispensable for the repression of chondrogenic marker genes in CM cells. Conditional deletion of β-catenin in the CM does not alter H3K27me3 enrichment around differentially expressed genes nor genome-wide *in vivo*. Our data in genetic mutants *in vivo* are consistent with a model whereby EZH2 and H3K27me3 are not required in the CM to guide cell fate selection. Interrogating mixed cell populations is unlikely to account for our major finding, given that our CM-restricted deletion of β-catenin did not lead to changes in H3K27me3 profiles, and *Ezh2* mutants *in vivo* did not show changes in cell fate selection in the supraorbital CM.

Considering that loss of β-catenin at E10.5 leads to ectopic chondrogenesis, but loss of *Ezh2* at E10.5 did not phenocopy the β-catenin mutant, the function of the physical interaction between β-catenin and PRC2 remains unclear. A recent study in human colon cancer cells demonstrated that EZH2 alone, independent of H3K27me3, was sufficient to repress transcription (O’Geen *et al.* 2017). While we did not observe genome-wide changes in H3K27me3 enrichment upon loss of β-catenin, it is possible that β-catenin is required to recruit EZH2 itself to the genome. Alternatively, EZH2 was recently shown to bind to β-catenin in mouse ESCs and trimethylate lysine 49 (K49me3) on the β-catenin protein itself (β-catMe3) (Hoffmeyer *et al.* 2017). The β-catMe3 protein could then function as a transcriptional repressor at defined loci in ESCs to govern neuronal *vs.* mesoderm fate. However, loss of *Ezh2* in the CM did not lead to alteration in cell fate selection, indicating that K49me3 modification of β-catenin does not play a role in cell fate selection in the CM. Future studies examining DNA binding by EZH2 and β-catenin could provide a biological function for the physical interaction between β-catenin and EZH2.

The lack of cell fate changes in the supraorbital CM of *Ezh2* mutants could indicate that the role of EZH2, and by extension PRC2, is dependent on the developmental stage and cell type. Most studies linking PRC2 and cell fate selection were performed in ESCs *in vitro*. Differences in the role of EZH2 between *in vivo* CM and *in vitro* ESCs may indicate that the cell fate selection role of PRC2 is unique to ES cells or linked to specific cell types. In addition, previous studies deleting *Ezh2* at similar developmental stages in the mouse embryo found varying craniofacial phenotypes and defects (Schwarz *et al.* 2014; Dudakovic *et al.* 2015). Deletion of *Ezh2* in the premigratory cranial neural crest cells with *Wnt1Cre* by E8.5 resulted in severe reduction of facial and skull bones, and embryonic lethality (Schwarz *et al.* 2014). Conditional deletion of *Ezh2* at E9.5 in posterior CM with *Prx1Cre* resulted predominantly in postnatal craniosynostosis. We did not find gross changes in embryonic craniofacial morphology upon deletion of *Ezh2* in the CM at E10.0 with *Dermo1Cre* (data not shown). These results suggest that the role of PRC2 in embryonic development may be cell type- and developmental stage-specific. Future studies *in vivo* are required to tease out the timing and dynamics of developmental gene regulation by PRC2.

There are recent data from several groups refining the role of PRC2 and H3K27me3 enrichment. According to the histone code hypothesis, H3K27me3 is often found on transcriptionally repressed genes and is widely considered to be a sign of transcriptional repression (Boyer *et al.* 2006; Lee *et al.* 2006; Roh *et al.* 2006; Barski *et al.* 2007; Heintzman *et al.* 2007, 2009). In early postmigratory mouse neural crest cells, H3K27me3 was shown to mark bivalent domains accessible by the activating mark H3K4me3, indicating transcriptional poising rather than repression (Minoux *et al.* 2017). Recently, the histone code model has been refined to show that H3K27me3 enrichment is not just predictive of transcriptional repression in mouse ESCs, but also indicative of a past transcriptional repressive state (Riising *et al.* 2014; Comet *et al.* 2016). Furthermore, in human colon cancer cells, ectopic deposition of H3K27me3 with an EZH2-dCas9 fusion construct was not sufficient for transcriptional repression (O’Geen *et al.* 2017). In mouse rib chondrocytes, an intersection of RNA-seq data with H3K27me3 ChIP-seq data also suggested that H3K27me3 enrichment on TSS was not sufficient for transcriptional repression. When compared to genes dysregulated upon knockout of a major PRC2 component, EED, only 11% of the genes dysregulated were enriched for H3K27me3. Thus, the biological role of the remaining 89% of H3K27me3 peaks is unclear (Mirzamohammadi *et al.* 2016). Our data are entirely consistent with these recent findings. Intersecting our *in vivo* RNA-seq and ChIP-seq studies revealed legitimate H3K27me3 peaks in genes that did not correlate with transcription levels. We found that both expressed and repressed genes in control CM+ectoderm can be enriched for H3K27me3, demonstrating that H3K27me3 is not sufficient to indicate repression. The H3K27me3 marks remain at *Sox9* and other cartilage marker genes in β-catenin mutants, suggesting that these marks may be carried over and reflective of a past transcriptional off state.

If PRC2 is not the principal repressor of chondrogenesis in CM, the question remains as to what factor exerts this function. We propose three other models that will require further testing. The first model calls for other epigenetic-related mechanisms such as direct covalent modifications of DNA (DNA methylation), or other histone modification-related mechanisms, such as G9a-associated K9me3 repression. A study in chick limb bud micromass cultures showed that the addition of exogenous Wnt3a led to an increase in DNA methylation by DNMT3a on the *Sox9* promoter (Kumar and Lassar 2014). However, in our hands, the addition of DNMT inhibitors did not alter *Sox9* and *Col2a1* mRNA levels in primary CM+ectoderm cells cultured *in vitro* (data not shown). Further studies *in vivo* will be required to investigate this model. The second model postulates that β-catenin activates yet-to-be-identified signaling pathways or transcription factors that would be directly involved in repression. For example, *Twist1* is positively regulated by Wnt/β-catenin signaling, and conditional deletion of *Twist1* partially phenocopies the ectopic chondrogenesis found in the *En1Cre/+*;*R26R/+*;β*-catenin^fl/^*^Δ^ mutants (Komori *et al.* 1997; Goodnough *et al.* 2012). The retinoic acid (RA) signaling pathway can interact with Wnt/β-catenin signaling, and it can promote chondrocyte development and function *in vitro* (Yasuhara *et al.* 2010; Uchibe *et al.* 2017). RA signaling pathway components are robustly expressed in the control CM. Their interaction with Wnt/β-catenin signaling and the role in the CM remains to be tested. A third model is that β-catenin does not directly control the transcription of cartilage determinants and marker genes, but may control the expression or activity of factors involved in the post-transcriptional modification of cell fate determination and chondrocyte differentiation genes.

Overall, our data suggested a model whereby the repression of the chondrogenic fate by Wnt/β-catenin signaling does not rely on EZH2 and H3K27me3, but implies other yet-to-be-identified transcriptional or post-transcriptional mechanisms.

## Supplementary Material

Supplemental material is available online at www.g3journal.org/lookup/suppl/doi:10.1534/g3.117.300311/-/DC1.
