# Supplementary material for: PRC2 Is Dispensable in Vivo for β-Catenin-Mediated Repression of Chondrogenesis in the Mouse Embryonic Cranial Mesenchyme
Source: G3 (Bethesda). 2017 Dec 9;8(2):491–503. doi: 10.1534/g3.117.300311 (PMC5919733; doi:10.1534/g3.117.300311)
Supplement: Supplementary file 1 [file 491FileS1.pdf]

Figure S1

A

	Twist1		*Twist2*		*Apcdd1*	
	En1Cre; β-catenin$^{fl/+}$	*En1Cre; β-catenin$^{fl/\Delta}$*	*En1Cre; β-catenin$^{fl/+}$*	*En1Cre; β-catenin$^{fl/\Delta}$*	*En1Cre; β-catenin$^{fl/+}$*	*En1Cre; β-catenin$^{fl/\Delta}$*
Average FPKM	64.32	23.51	38.21	6.65	45.21	10.03
Standard Deviation	8.50	3.36	4.32	0.92	4.46	2.47

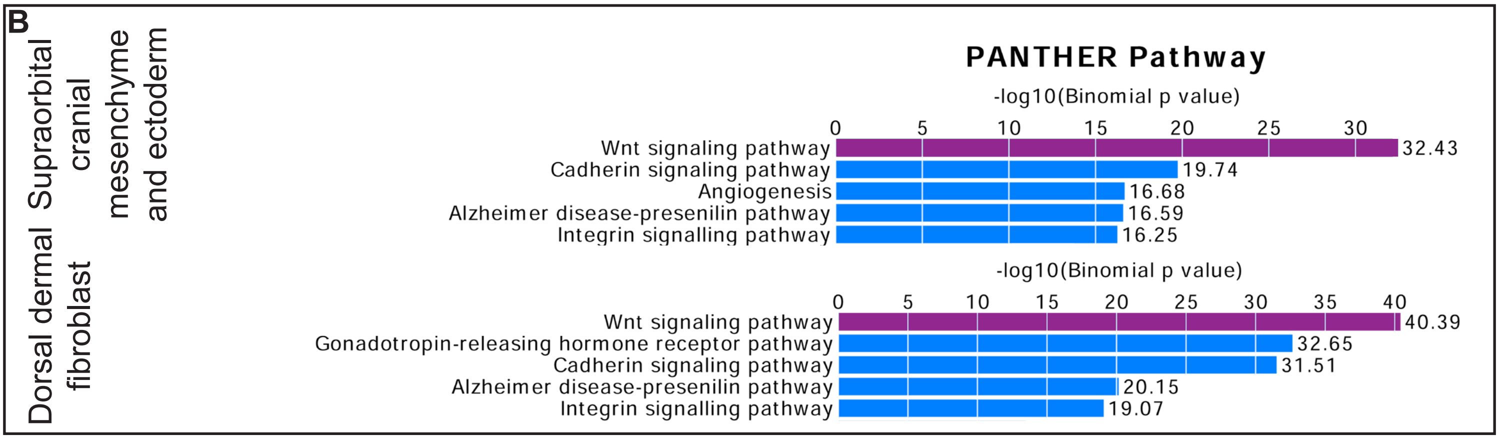

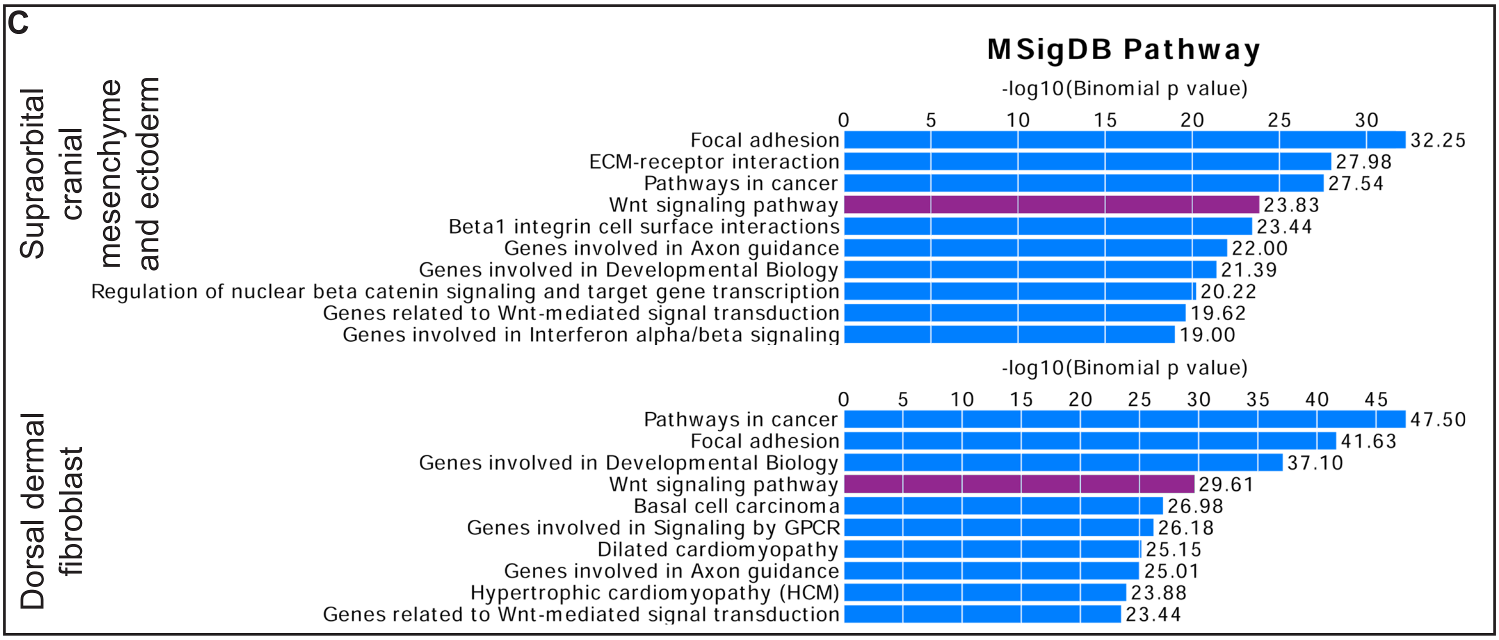

Figure S2

A

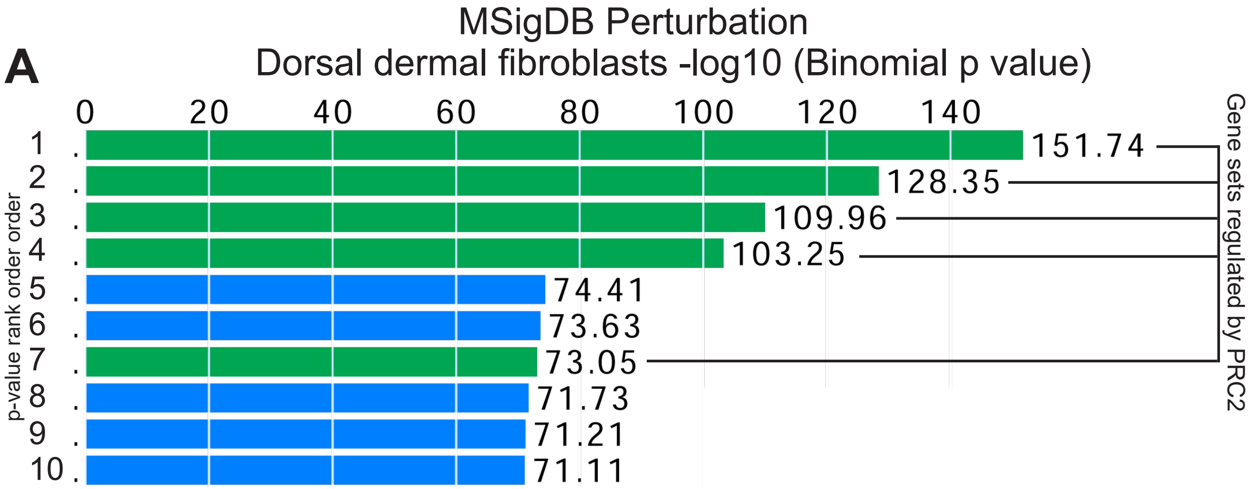
MSigDB Perturbation
Dorsal dermal fibroblasts -log10 (Binomial p value)
0
20
40
60
80
100
120
140
p-value rank order order
1 .
2 .
3 .
4 .
5 .
6 .
7 .
8 .
9 .
10 .
151.74
128.35
109.96
103.25
74.41
73.63
73.05
71.73
71.21
71.11
Gene sets regulated by PRC2

B

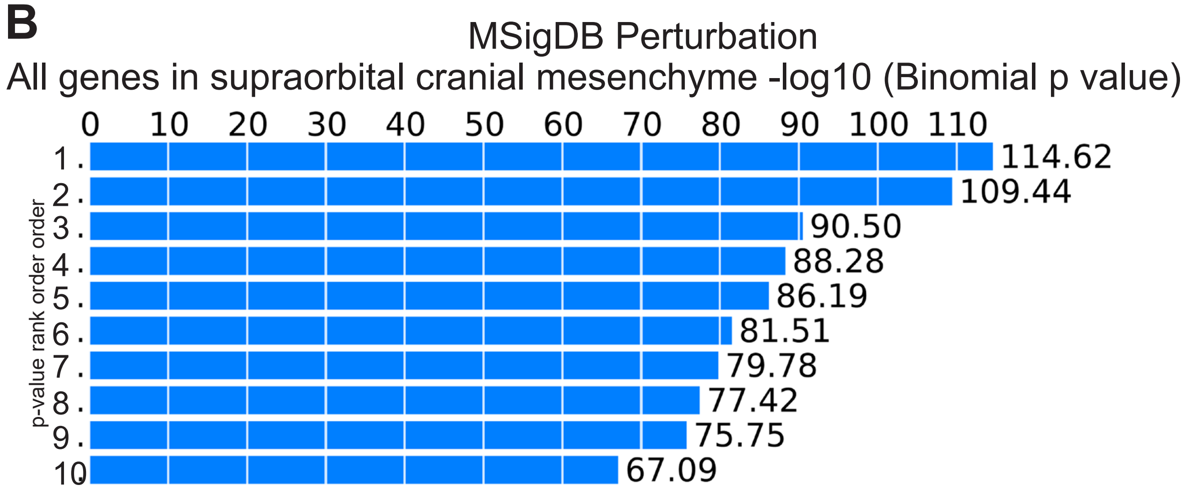
MSigDB Perturbation
All genes in supraorbital cranial mesenchyme -log10 (Binomial p value)
0
10
20
30
40
50
60
70
80
90
100
110
p-value rank order order
1 .
2 .
3 .
4 .
5 .
6 .
7 .
8 .
9 .
10
114.62
109.44
90.50
88.28
86.19
81.51
79.78
77.42
75.75
67.09

Figure S3

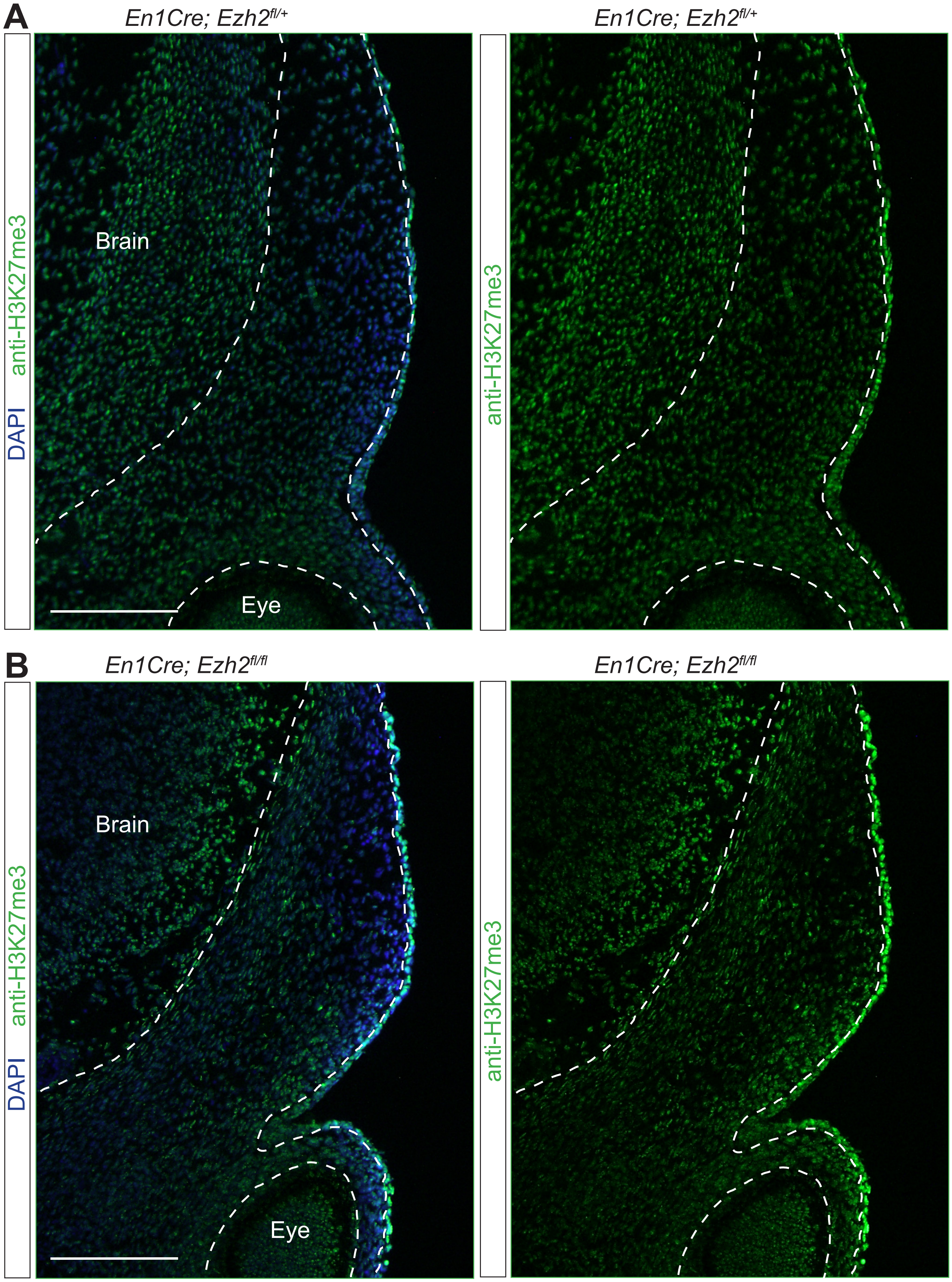
A
En1Cre; Ezh2fl/+
En1Cre; Ezh2fl/+
DAPI anti-H3K27me3
anti-H3K27me3
Brain
Eye
B
En1Cre; Ezh2fl/fl
En1Cre; Ezh2fl/fl
DAPI anti-H3K27me3
anti-H3K27me3
Brain
Eye

Figure S4

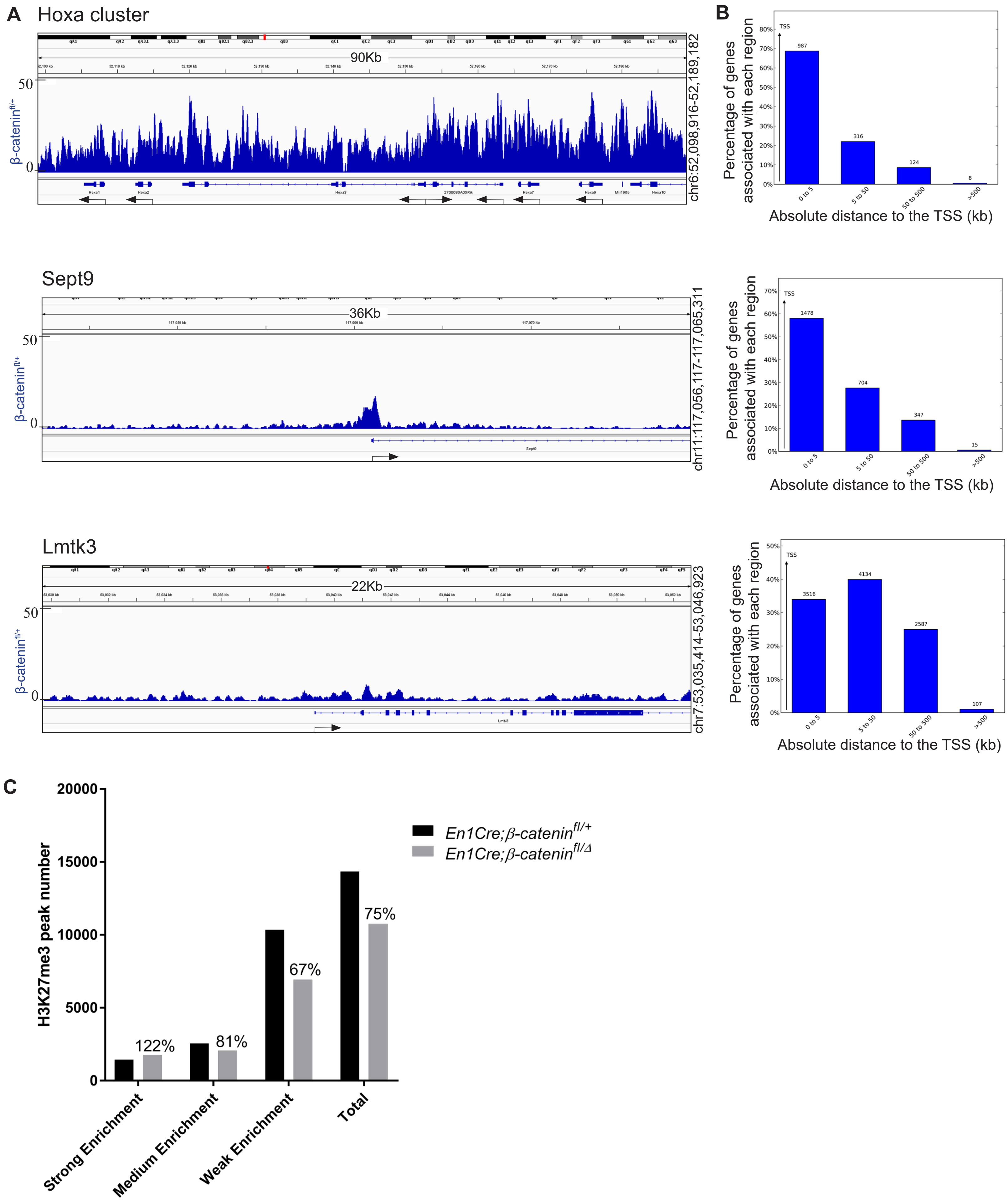

A
Hoxa cluster
90Kb
50
0
β-catenin fl/+
chr6:52,098,916-52,189,182
Sept9
36Kb
chr11:117,056,117-117,065,311
Lmtk3
22Kb
chr7:53,035,414-53,046,923
B
Percentage of genes associated with each region
Absolute distance to the TSS (kb)
TSS
987
316
124
8
1478
704
347
15
3516
4134
2587
107
0 to 5
5 to 50
50 to 500
>500
C
H3K27me3 peak number
20000
15000
10000
5000
0
En1Cre;β-catenin fl/+
En1Cre;β-catenin fl/Δ
122%
81%
67%
75%
Strong Enrichment
Medium Enrichment
Weak Enrichment
Total

Figure S5

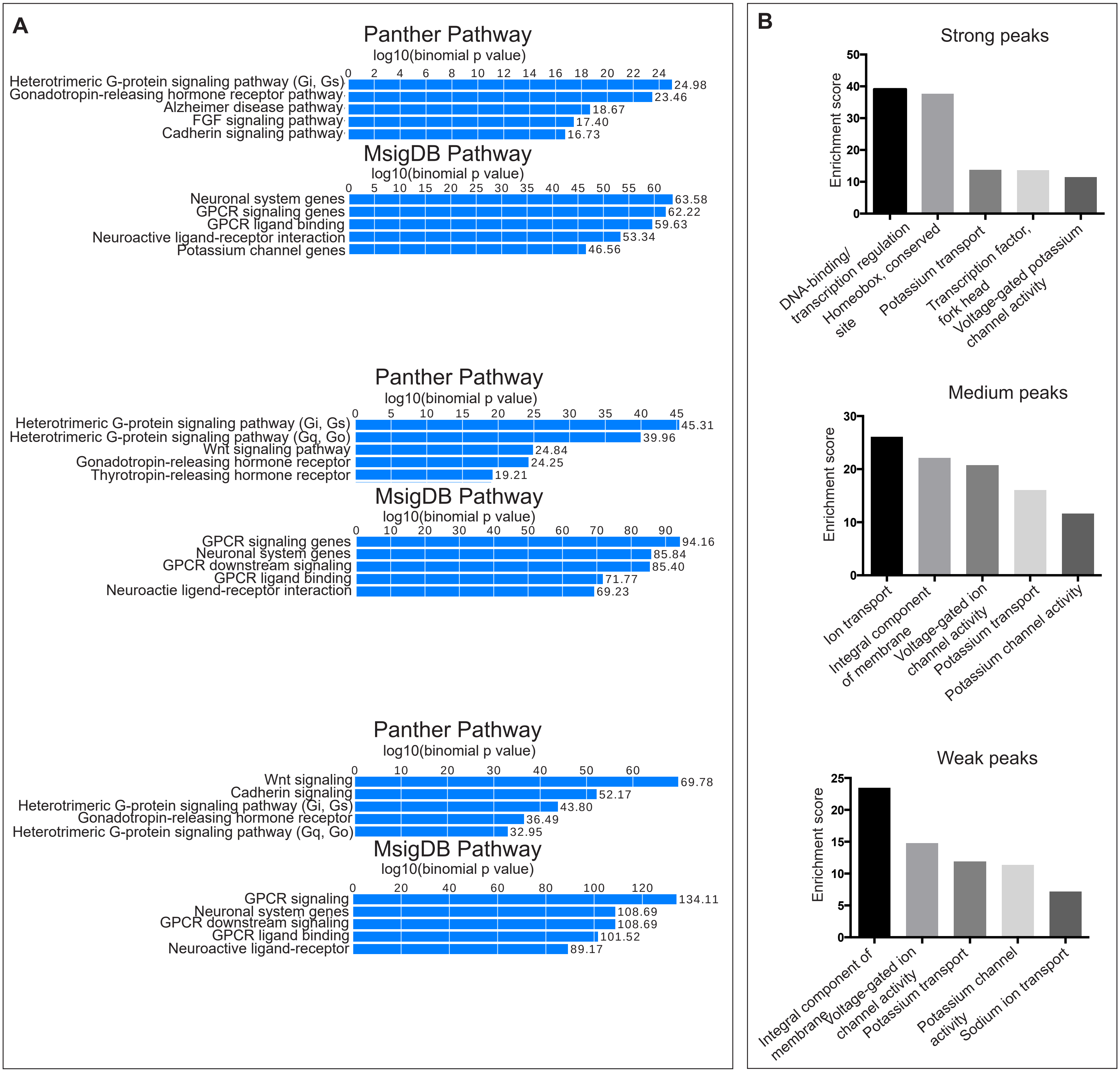

A
Panther Pathway
log10(binomial p value)
0 2 4 6 8 10 12 14 16 18 20 22 24
Heterotrimeric G-protein signaling pathway (Gi, Gs) 24.98
Gonadotropin-releasing hormone receptor pathway 23.46
Alzheimer disease pathway 18.67
FGF signaling pathway 17.40
Cadherin signaling pathway 16.73
MsigDB Pathway
log10(binomial p value)
0 5 10 15 20 25 30 35 40 45 50 55 60
Neuronal system genes 63.58
GPCR signaling genes 62.22
GPCR ligand binding 59.63
Neuroactive ligand-receptor interaction 53.34
Potassium channel genes 46.56
Panther Pathway
log10(binomial p value)
0 5 10 15 20 25 30 35 40 45
Heterotrimeric G-protein signaling pathway (Gi, Gs) 45.31
Heterotrimeric G-protein signaling pathway (Gq, Go) 39.96
Wnt signaling pathway 24.84
Gonadotropin-releasing hormone receptor 24.25
Thyrotropin-releasing hormone receptor 19.21
MsigDB Pathway
log10(binomial p value)
0 10 20 30 40 50 60 70 80 90
GPCR signaling genes 94.16
Neuronal system genes 85.84
GPCR downstream signaling 85.40
GPCR ligand binding 71.77
Neuroactie ligend-receptor interaction 69.23
Panther Pathway
log10(binomial p value)
0 10 20 30 40 50 60
Wnt signaling 69.78
Cadherin signaling 52.17
Heterotrimeric G-protein signaling pathway (Gi, Gs) 43.80
Gonadotropin-releasing hormone receptor 36.49
Heterotrimeric G-protein signaling pathway (Gq, Go) 32.95
MsigDB Pathway
log10(binomial p value)
0 20 40 60 80 100 120
GPCR signaling 134.11
Neuronal system genes 108.69
GPCR downstream signaling 108.69
GPCR ligand binding 101.52
Neuroactive ligand-receptor 89.17
B
Strong peaks
Enrichment score
0 10 20 30 40 50
DNA-binding/ transcription regulation
Homeobox, conserved site
Potassium transport
Transcription factor, fork head
Voltage-gated potassium channel activity
Medium peaks
Enrichment score
0 10 20 30
Ion transport
Integral component of membrane
Voltage-gated ion channel activity
Potassium transport
Potassium channel activity
Weak peaks
Enrichment score
0 5 10 15 20 25
Integral component of membrane
Voltage-gated ion channel activity
Potassium transport
Potassium channel activity
Sodium ion transport

Figure S6

A

H3K27me3 ChIP sequencing peak strength	Total Associated Genes	Associated with cranial mesenchyme genes >1 FPKM	Percentage	Associated with cranial mesenchyme genes < 1FPKM	Percentage
Strong Peaks	891	224	25.14	628	70.48
Medium Peaks	1269	524	41.29	676	53.27
Weak Peaks	2107	945	44.85	1011	47.98

B

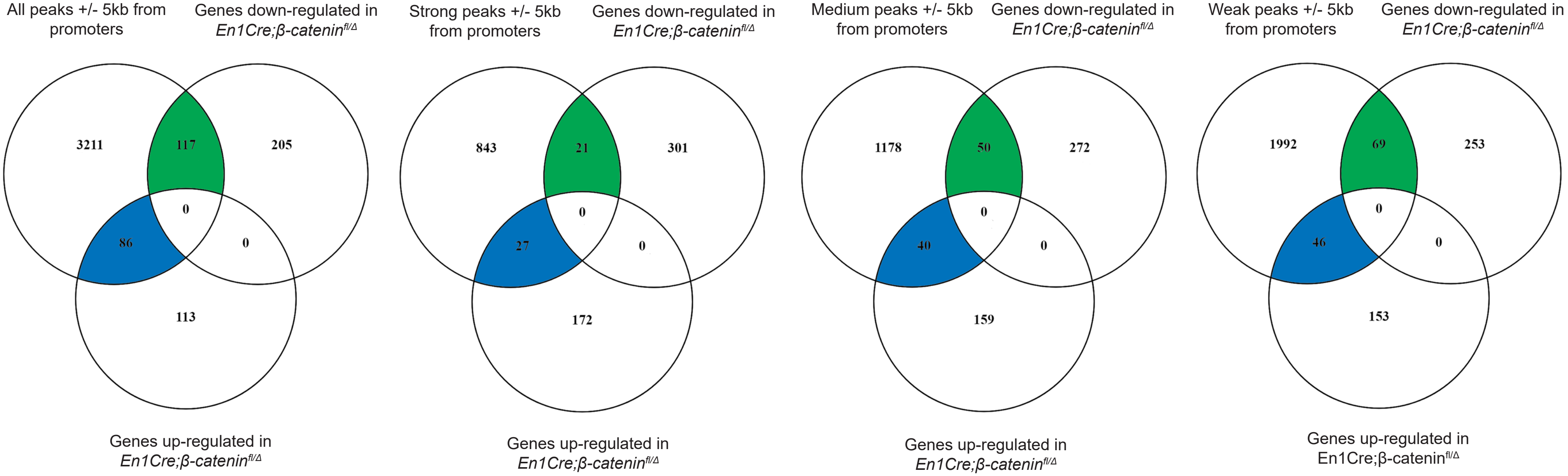

Figure S7

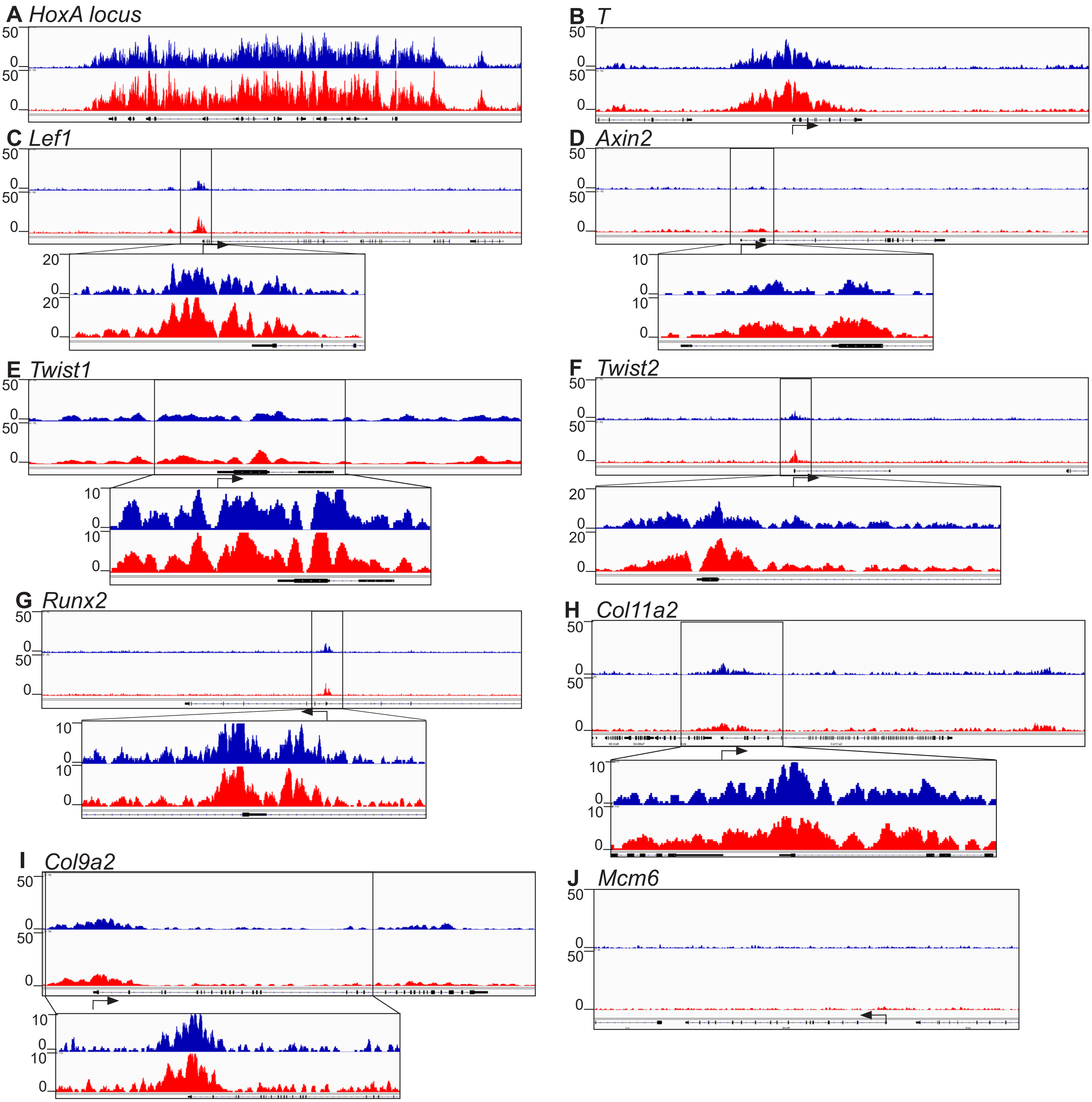
A HoxA locus
B T
C Lef1
D Axin2
E Twist1
F Twist2
G Runx2
H Col11a2
I Col9a2
J Mcm6
50
0
20
10

Table1

INTERACTION	TISSUE	TREATMENT	METHOD	BIOLOGICAL SIGNIFICANCE	AUTHOR
β-catenin and EZH2	MCF-7 human breast cancer cells	n/a	β-catenin and EZH2 co-IP	[do no show]	Shi et al., Mol and Cell Bio 2007
β-catenin and EZH2	4-month old primary mouse mammay epithelium	EZH2 overexpression cassette injected into one cell zygotes	β-catenin and EZH2 co-IP and immunohistochemistry	EZH2 binds to β-catenin and induces nuclear accumulation	Li et al., American J of Pathology 2009
Wnt/β-catenin signaling and EZH2	Mouse brown fat	adCre infected EZH2fl/fl in primary culture	qPCR (Wnts) and western blot (β-catenin), EZH2 ChIP-qPCR	EZH2 knockout leads to an up-regulation of Wnt1, 6, 10a, and 10b along with increased cytosolic β-catenin. EZH2 binds to Wnt1, 6, 10a, and 10b, but not β-catenin	Wang et al., Proceedings of National Academy of Science of the USA 2010
β-catenin and EZH2	HeLa cells	n/a	Co-IP	Enhances Wnt transactivation independent of EZH2 methylation activity	Jung et al., Mol Cell 2013
TCF3 affects EZH2 expression	Zebrafish retina	Non-functional TCF3 transgene	EZH2 and Suz12 *in situ* hybridization	Decrease in EZH2 expression in zebrafish retina	Aldiri et al., Development 2013)
Wnt3a and H3K27me3	4 day old chick limb bud micromass culture	Addition of Wnt3a in culture	ChIP-qPCR	Addition of Wnt3a increased H3K27me3 enrichment on the *Sox9* promoter	Kumar et al., Cell Reports 2014
Wnt signaling and EZH2	10T 1/2 cells	Addition of BMP4 in culture	ChIP-qPCR	BMP4 induces EZH2 and H3K27me3 enrichment on Wnt6, Wnt10a, and Wnt10b	Yi et al., Molecular Cell 2015
Wnt signaling and EZH2	E10.5 mouse midbrain	Wnt1Cre;EZH2fl/fl	E10.5 Wnt1Cre;EZH2fl/fl midbrains microarray and RT-qPCR	Knockout of EZH2 in midbrain leads to an up-regulation of Wnt inhibitors DKK2 and Wif1	Zemke et al., BMC Biology 2015
β-catenin and Jarid2	Mouse embryonic stem cells	Jarid2-/-	β-catenin western blot	Jarid2-/- leads to reduced β-catenin activity	Landeira et al., Cell Reports 2015
Wnt signaling and EZH2 and EZH1	3 day old tibial growth plate	Col2Cre;EZH1/2fl/fl	Microarray	Wnt signaling genes (Fzd6, Plcb1, Nfatc4, Wnt5a) are differentially expressed in deletion of EZH1/2.	Liu et al., Nature Communications 2016
Wnt signaling and EED	Neonatal tibial rib chondrocyte cultures	Col2Cre;EEDfl/fl	TopFlash and Wnt inhibitor	EED knockout out increases Wnt3a resposiveness by TopFlash. Administration of Wnt inhibitor, C59, rescues EED knockout kyphosis phenotype.	Mirzamohammadi et al., Nat Comm 2016
β-catenin and EZH2	Mouse embryonic stem cells	n/a	multiple experiments	EZH2 binds to β-catenin and induces nuclear accumulation	Hoffmeyer et al., Cell Reports 2017
