# Supplementary material for: PRC2 Is Dispensable in Vivo for β-Catenin-Mediated Repression of Chondrogenesis in the Mouse Embryonic Cranial Mesenchyme
Source: G3 (Bethesda). 2017 Dec 9;8(2):491–503. doi: 10.1534/g3.117.300311 (PMC5919733; doi:10.1534/g3.117.300311)
Supplement: Supplementary file 2 [file 491FileS2.docx]

**Supplementary Figure 1:**

**Genomic Regions Enrichment of Annotations Tool (GREAT) analysis of differentially expressed genes upon deletion of β-catenin.**

(A) Identification of expected gene expression changes between *En1Cre/+;R26R/+;β-catenin^fl/∆^* mutant and *En1Cre/+;R26R/+;β-catenin^fl/+^* control RNA-seq. (B) The top biological pathways represented in the differentially expressed genes in *En1Cre/+;R26R/+;β-catenin^fl/∆^* mutant and *En1Cre/+;R26R/+;β-catenin^fl/+^* control tissue. (B) The top biological pathways from the Panther Pathway gene ontology. (C) The top biological pathways from the MsigDB ontology. All ontologies ranked by binomial P-value.

**Supplementary Figure 2: Genes regulated by β-catenin are enriched for PRC2 targets in dorsal dermal fibroblasts.** (A) Differentially expressed genes in *En1Cre/+;R26R/+;β-catenin^fl/∆^* mutant and *En1Cre/+;R26R/+;β-catenin^fl/+^* control dorsal dermal fibroblasts were run through GREAT. Overlapping gene sets from the MsigDB Perturbations ontology were ranked by binomial P-value. Green bars represent gene sets regulated by PRC2. The ranked list is as follows: 1) Genes with CpG enriched promoters bearing H3K4me2 and H3K27me3 in brain. 2) Genes up-regulated in ES (human embryonic stem cells) with deficient SUZ12 [GeneID=23512]. 3) Genes up-regulated in TIG3 cells (human fibroblasts) upon knockdown of EED [GeneID=8726]. 4) H3K27me3 target genes in hESC identified by ChIP on chip. 5) Genes up-regulated in the normal-like subtype of breast cancer. 6) Genes up-regulated in *in vitro* stromal cells from adipose tissue compared to *in vivo* cells. 7) Genes possessing H3K27me3, SUZ12 [GeneID=23512], and EED [GeneID=8726] in hESC by ChIP on ChIP. 8) Genes coordinately up-regulated in a compendium of adult tissue stem cells. 9) Genes up-regulated in uterus upon knockout of BMP2 [GeneID=650]. 10) Genes consistently up-regulated in mammary cells in both humans and mice. (B) All genes identified in the *En1Cre/+;R26R/+;β-catenin^fl/+^* CM+ectoderm were run through GREAT. The ranked list is as follows: 1) Genes down-regulated in NB4 cells (acute promyelocytic leukemia, APL) in response to tretinoin [PubChem=444795]. 2) Genes with promoters bound by E2F4 [GeneID=1874] in unstimulated hybridoma cells. 3) Genes up-regulated in ME-A cells (breast cancer) undergoing apoptosis in response to doxorubicin [PubChem=31703]. 4) Genes whose promoters are bound by MYC [GeneID=4609]. 5) Up-regulated genes in colon carcinoma tumors compared to the matched normal mucosa samples. 6) Targets of c-Myc [GeneID=4609] and Max [GeneID=4149] identified by ChIP on chip in a Burkitt's lymphoma cell line. 7) Genes up-regulated in liver tumor compared to the normal adjacent tissue. 8) Genes up-regulated in lymphoblastoid cells from the European population compared to those from the Asian population. 9) Genes up-regulated in robust Cluster 2 (rC2) of hepatoblastoma samples compared to those in the robust Cluster 1 (rC1). 10) Housekeeping genes identified as expressed across 19 normal tissues.

**Supplementary Figure 3: Knockout of *Ezh2* with *En1Cre* does not lead to a change in H3K27me3 enrichment in the CM.** Indirect immunofluorescence in *En1Cre;Ezh2^fl/+^* and *En1Cre;Ezh2^fl/fl^* supraorbital CM of the PRC2 repressive mark H3K27me3. No qualitative difference in between (A) *En1Cre;Ezh2^fl/+^* and (B) *En1Cre;Ezh2^fl/fl^* is observed. Scale bars: 200µm.

**Supplementary Figure 4: Analysis of strong, medium, and weak H3K27me3 enrichment in the CM+ectoderm.** H3K27me3 ChIP-sequencing reads were grouped based on fold enrichment (strong peaks = >20, medium peaks = 10-20, weak peaks = <10), and were mapped to the single nearest gene using GREAT. (A) IGV representation of each class of peak. (B) Distribution of H3K27me3 strong, medium, and weak peaks in relation to the transcription start site of the single nearest gene to the peak.  (C) Number of peaks in each class between *En1Cre/+;R26R/+;β-catenin^fl/+^* controls and *En1Cre/+;R26R/+;β-catenin^fl/∆^* mutants.

**Supplementary Figure 5: Gene ontology analysis of strong, medium, and weak H3K27me3 enrichment.** Gene ontology analysis on a list of genes pertaining to each class of H3K27me3 peaks generated by GREAT. The gene set is limited to include only the genes with a H3K27me3 peak +/- 5kb from their transcription start site. (A) Top five biological pathways identified in the Panther and MsigDB ontology databases for genes containing each class of peaks. Analysis was performed using GREAT.  (B) DAVID ontology analysis of the gene list from each class of H3K27me3 peak.

**Supplementary Figure 6: Strong, medium, and weak H3K27me3 peak groups have similar representation in genes regulated by β-catenin.** Intersection of *En1Cre/+;R26R/+;β-catenin^fl/+^* control and *En1Cre/+;R26R/+;β-catenin^fl/∆^* mutant RNA-seq data with genes bound each class of H3K27me3 peak. (A) Number of each class of peak found in all expressed genes (>1 FPKM) and repressed genes (<1 FPKM) identified in *En1Cre/+;R26R/+;β-catenin^fl/+^* control CM+ectoderm. (B) Overlap of genes up- and down-regulated in *En1Cre/+;R26R/+;β-catenin^fl/∆^* mutants with all, strong, medium, and weak peaks.

**Supplementary Figure 7: H3K27me3 peaks are associated with genes actively transcribed and repressed in CM.** Integrated genome viewer representation of H3K27me3 peaks in *En1Cre/+;R26R/+;β-catenin^fl/+^* control and *En1Cre/+;R26R/+;β-catenin^fl/∆^* mutant CM+ectoderm. (A-B) The HoxA locus and *T* are known targets of PRC2. (C-F) *Lef1, Axin2, Twist1*, and *Twist2* are down-regulated upon the deletion of β-catenin. (G) *Runx2* is an osteoblast marker. (H,I) *Col11a2* and *Col9a2* are chondrocyte markers. (J) *Mcm6* is not bound by H3K27me3 in both the controls and mutants.

**Supplementary Table 1:**

Summary of publications demonstrating a biological interaction between β-catenin and PRC2.
